# Human Onchocerciasis: Modelling the Potential Long-term Consequences of a Vaccination Programme

**DOI:** 10.1371/journal.pntd.0003938

**Published:** 2015-07-17

**Authors:** Hugo C. Turner, Martin Walker, Sara Lustigman, David W. Taylor, María-Gloria Basáñez

**Affiliations:** 1 London Centre for Neglected Tropical Disease Research, Department of Infectious Disease Epidemiology, School of Public Health, Faculty of Medicine (St. Mary’s Campus), Imperial College London, London, United Kingdom; 2 Department of Infectious Disease Epidemiology, School of Public Health, Faculty of Medicine (St. Mary’s Campus), Imperial College London, London, United Kingdom; 3 Laboratory of Molecular Parasitology, Lindsley F. Kimball Research Institute, New York Blood Center, New York, New York, United States of America; 4 Division of Infection and Pathway Medicine, University of Edinburgh Medical School, The Chancellor’s Building, Edinburgh, United Kingdom; University of Liverpool, UNITED KINGDOM

## Abstract

**Background:**

Currently, the predominant onchocerciasis control strategy in Africa is annual mass drug administration (MDA) with ivermectin. However, there is a consensus among the global health community, supported by mathematical modelling, that onchocerciasis in Africa will not be eliminated within proposed time frameworks in all endemic foci with only annual MDA, and novel and alternative strategies are urgently needed. Furthermore, use of MDA with ivermectin is already compromised in large areas of central Africa co-endemic with *Loa loa*, and there are areas where suboptimal or atypical responses to ivermectin have been documented. An onchocerciasis vaccine would be highly advantageous in these areas.

**Methodology/Principal Findings:**

We used a previously developed onchocerciasis transmission model (EPIONCHO) to investigate the impact of vaccination in areas where loiasis and onchocerciasis are co-endemic and ivermectin is contraindicated. We also explore the potential influence of a vaccination programme on infection resurgence in areas where local elimination has been successfully achieved. Based on the age range included in the Expanded Programme on Immunization (EPI), the vaccine was assumed to target 1 to 5 year olds. Our modelling results indicate that the deployment of an onchocerciasis vaccine would have a beneficial impact in onchocerciasis–loiasis co-endemic areas, markedly reducing microfilarial load in the young (under 20 yr) age groups.

**Conclusions/Significance:**

An onchocerciasis prophylactic vaccine would reduce the onchocerciasis disease burden in populations where ivermectin cannot be administered safely. Moreover, a vaccine could substantially decrease the chance of re-emergence of *Onchocerca volvulus* infection in areas where it is deemed that MDA with ivermectin can be stopped. Therefore, a vaccine would protect the substantial investments made by present and past onchocerciasis control programmes, decreasing the chance of disease recrudescence and offering an important additional tool to mitigate the potentially devastating impact of emerging ivermectin resistance.

## Author Summary

Novel and alternative strategies are required to meet the demanding control and elimination (of infection) goals for human onchocerciasis (river blindness) in Africa. Due to the overlapping distribution of onchocerciasis and loiasis (African eye worm) in forested areas of central Africa, millions of people living in such areas are not well served by current interventions because they cannot safely receive the antiparasitic drug ivermectin that is distributed *en masse* to treat onchocerciasis elsewhere in Africa. The Onchocerciasis Vaccine for Africa—TOVA—Initiative has been established to develop and trial an onchocerciasis vaccine. We model the potential impact of a hypothetical childhood vaccination programme rolled out in areas where co-endemicity of onchocerciasis and African eye worm makes mass distribution of ivermectin difficult and potentially unsafe for treating, controlling and eliminating river blindness. We find that, 15 years into the programme, a vaccine would substantially reduce infection levels in children and young adults, protecting them from the morbidity and mortality associated with onchocerciasis. Most benefit would be reaped from a long-lived vaccine, even if only partially protective. We also discuss how a vaccine could substantially reduce the risk of re-emergence of onchocerciasis in areas freed from infection after years of successful intervention.

## Introduction

Currently, the predominant onchocerciasis control strategy in Africa is annual mass drug administration (MDA) with ivermectin, which Merck & Co. have committed to donate for as long as needed to eliminate onchocerciasis as a public health problem. Since 2010 there has been a dramatic shift in onchocerciasis control policy in Africa, with programmes changing their aim from elimination of the disease burden to elimination of the infection where feasible. The World Health Organization’s (WHO) Roadmap on Neglected Tropical Diseases [1]—endorsed by the London Declaration on NTDs (LDNTD, 31 January 2012) [2]—set goals for elimination of *Onchocerca volvulus* infection in selected countries of Africa by 2020. The African Programme for Onchocerciasis Control (APOC) has pledged elimination of onchocerciasis where possible by 2025 [3], and the Bill and Melinda Gates Foundation foresees that global elimination will be reached by 2030 [4]. We have previously indicated, based on mathematical modelling of onchocerciasis transmission and control with EPIONCHO, that the feasibility of eliminating the infection depends primarily on baseline (pre-control) levels of endemicity, patterns of transmission, magnitude of residual transmission between inter-treatment periods, therapeutic coverage and importantly, compliance to treatment, precluding a one-size-fits-all approach to elimination [5,6,7,8]. There is a consensus among the global health community, substantiated by mathematically modelling, that onchocerciasis in Africa will not be eliminated in all endemic foci with annual ivermectin MDA alone [9,10,11], and that novel supportive health intervention technologies, including a vaccine, and/or alternative treatment and control strategies are badly needed [2,12,13].

Mass distribution of ivermectin is already compromised in large areas of central Africa (including the Congo basin) [14], where another filarial infection, loiasis or eye-worm, is co-endemic with human onchocerciasis and ivermectin cannot be used for the treatment of individuals with high *Loa loa* microfilaraemia (microfilariae in blood) because of the risk of developing severe and possibly fatal or irreversible adverse reactions [15,16]. Currently, it is recommended that in areas co-endemic for these two filarial infections, where *L*. *loa* microfilarial prevalence is above a threshold of 20% [15], ivermectin should not be distributed [16] as there is an unacceptable risk of severe adverse events (SAEs). It has been estimated that approximately 14 million people live in high-risk loiasis areas in central Africa and are potentially affected by this contraindication [14]. However, the true extent of the overlap between onchocerciasis and loiasis, as well as the levels of infection prevalence and intensity for both infections and of *L*. *loa* microfilarial load in co-infected individuals within such co-endemic areas need to be ascertained [17].

Stopping ivermectin treatment following local elimination of infection brings the inescapable risk of infection recrudescence seeded by migrating and infective blackflies and/or humans from areas with ongoing transmission. Modelling has shown that the time to reach elimination varies considerably with the intensity of transmission, taking longer in high endemicity areas compared to low endemicity areas [5,6,9]. Hence, it is likely that the highest endemicity areas with the most intense transmission will become sources of infection to an increasing number of infection-free communities as progress towards global elimination goals advances.

In addition to the above considerations, suboptimal or atypical responses to ivermectin have been documented in some communities, particularly in Ghana where mass ivermectin distribution first started. These responses manifest as a faster than anticipated rate of microfilarial reappearance in the skin following treatment [18,19,20,21]. This has raised concerns that the parasite may be developing incipient resistance to the embryostatic effect of ivermectin [18,19,20,21]. If ivermectin resistance were to develop, it could eventually spread and the likelihood of onchocerciasis elimination by MDA with ivermectin as a stand-alone strategy would be jeopardised.

The Onchocerciasis Vaccine for Africa (TOVA) initiative is a response to the demand for new intervention tools for onchocerciasis control and elimination [13,22,23]. TOVA builds upon over 30 years of research aimed at developing and testing an *O*. *volvulus* vaccine, a project initiated by the Edna McConnell Clark Foundation (1985–1999) [24,25] and subsequently supported by the European Union and the National Institutes for Health of the USA. TOVA has identified three prime vaccine candidates (*Ov*-103, *Ov*-RAL-2, and *Ov*-CPI-2M) based on proven efficacy in animal model systems [22,26,27,28,29,30], aiming to take at least one of these experimental vaccines to phase II efficacy trials by 2020 [22].

Here, we extend a previously developed onchocerciasis dynamic transmission model to: (a) investigate the potential impact of vaccination in areas where ivermectin is contraindicated because of onchocerciasis–loiasis co-endemicity, and (b) explore its potential influence on infection resurgence in controlled areas.

## Methods

### Model

**Fig 1:**
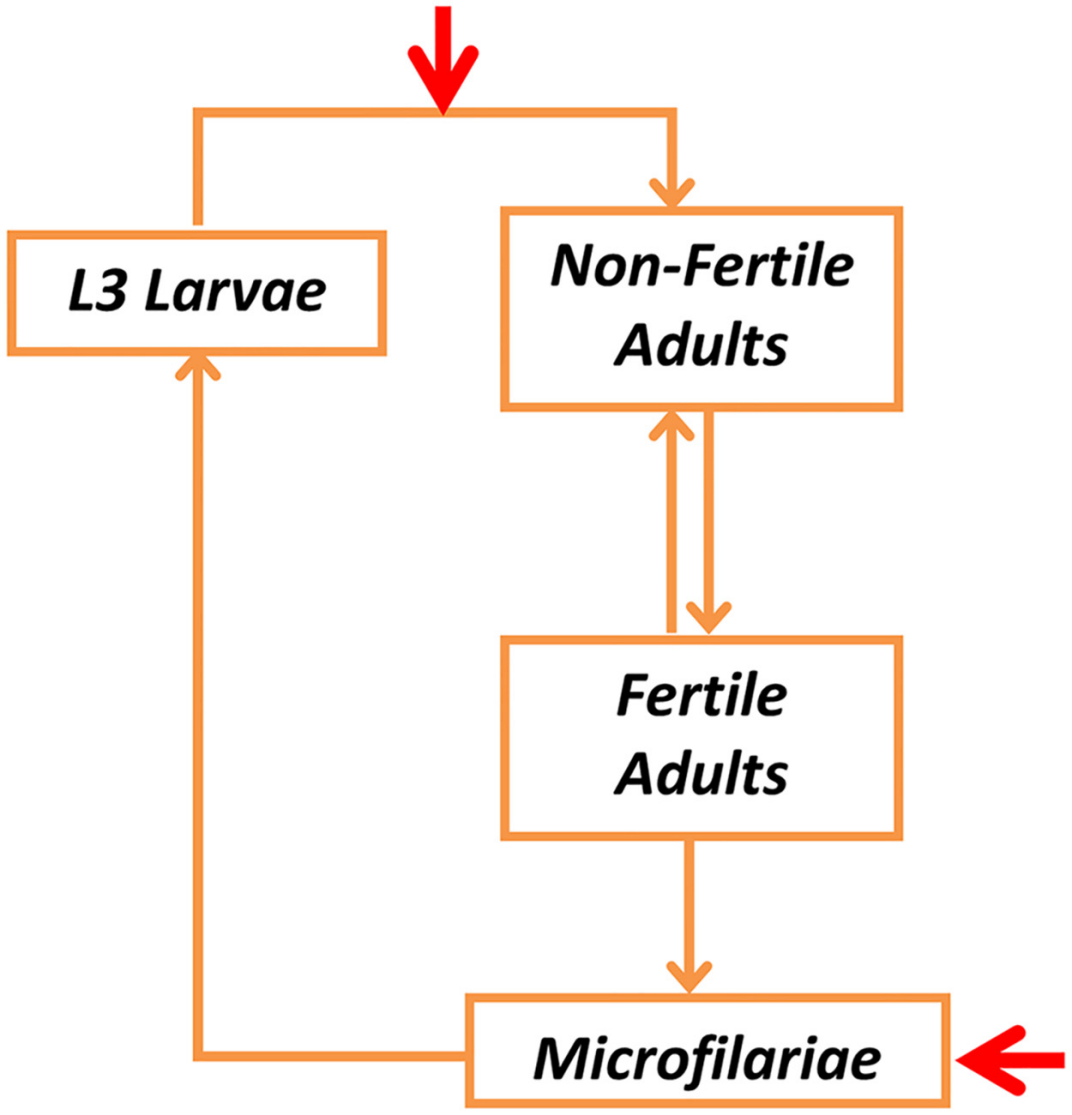
Schematic representation of EPIONCHO. The red arrows indicate the points in the *Onchocerca volvulus* lifecycle on which a hypothetical vaccine is assumed to have an effect; namely on parasite establishment and microfilariae.

The analysis was performed using our deterministic onchocerciasis transmission model (EPIONCHO) which describes the rate of change with respect to time and host age (in both sexes) of the mean number of fertile and non-fertile female adult worms per host, the mean number of microfilariae (mf) per milligram (mg) of skin, and the mean number of L3 larvae per simuliid fly (Fig 1). The model has been refined from the original framework developed by Basáñez and Boussinesq [31], to include age and sex structure of the host population [32]; the population-level effects of a single [33,34] and multiple [8] treatments with ivermectin, and increased programmatic realism related to patterns of treatment coverage and systematic non-compliance (whose effects can be explored separately) [8].

**Fig 2:**
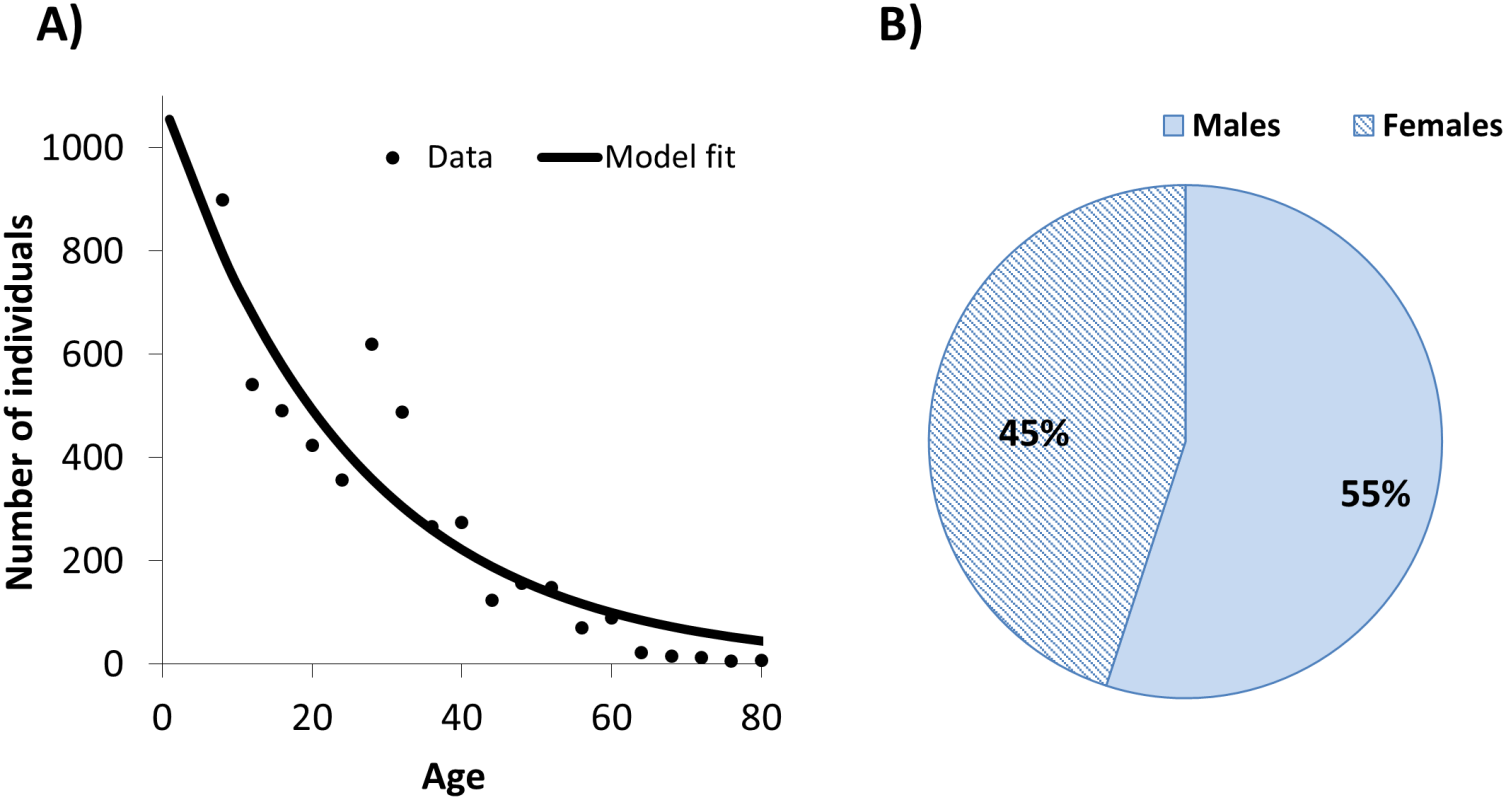
EPIONCHO’s underlying demography. **(A)** Age distribution and **(B)** Human sex ratio parameterised for savannah settings of northern Cameroon [32,35,36].

The assumed human age- and sex-structure of the population reflects demographic characteristics in savannah areas of northern Cameroon [32,35,36] (Fig 2), where the prevailing *O*. *volvulus–Simulium damnosum sensu lato* combinations (i.e. savannah parasites–*S*. *damnosum sensu stricto* /*S*. *sirbanum*) are responsible for the most severe sequelae of onchocerciasis [37,38]. The model captures age- and sex-specific host exposure to biting blackfly vectors (Fig 2A), calibrated to reproduce observed pre-control microfilarial load (infection intensity) age profiles (Fig 2B) in Cameroon [32], epidemiological patterns which are also seen in forest areas of Cameroon [35] and elsewhere in foci under vector control in the Onchocerciasis Control Programme in West Africa (OCP) area [39]. We assumed a stationary age distribution and a stable (closed) population. The model can reflect pre-control infection levels in a range of hypo-, meso-, hyper- and highly hyperendemic onchocerciasis foci (Table 1) by varying the annual biting rate (ABR) of the simuliid vectors (the number of bites received per person per year). A more detailed explanation of the model is provided in S1 File (Text A, Text B, Table A, Table B and Table C).

**Table 1:** Endemicity categories as defined by microfilarial prevalence.

Endemicity	Microfilarial prevalence
Hypoendemic	<35%
Mesoendemic	35% to 60%
Hyperendemic	Over 60%
Highly hyperendemic	Over 80%

Values adapted from [72].

### Vaccine Effects

Our extended version of EPIONCHO assumes that the vaccine exerts two effects (Fig 1), a prophylactic effect against incoming L3 (infective) stage-larvae and a therapeutic effect against mf (the stage responsible for transmission to vectors and onchocercal pathology). These effects—which are represented phenomenologically rather than mechanistically—are assumed to manifest, respectively, as a proportional reduction in the probability that an incoming L3 larva develops into a reproductively functional adult worm (prophylactic effect), and as a proportional reduction in the skin microfilarial load (therapeutic effect) (S1 File, Text C).

Based on animal model data [26,27,28], we assumed an initial prophylactic efficacy of 50%, and an initial therapeutic efficacy of 90%. We also explored higher initial vaccine efficacies of, respectively, 70% and 95% in a sensitivity analysis. We assumed that these initial prophylactic and therapeutic effects wane at a rate of 0.05 per year such that their mean duration is 20 years (= 1 / 0.05). As part of our sensitivity analysis, we varied this rate of decay (mean duration between 5 and 50 years), in accordance with the range considered by previous modelling of a hypothetical schistosomiasis vaccine [40].

We modelled a vaccination programme targeting one- to five-year olds in its first year, representing an initial ‘catch-up’ campaign, followed by continuous vaccination of one-year olds subsequently (hence each child would receive a single vaccination, as they become 1). This was based on the age range included in the Expanded Programme on Immunization (EPI) [41]. We also considered a less intensive alternative programme, omitting the initial ‘catch-up’ component, and involving the vaccination of five-year olds only. A compromised schedule such as this could be necessary given the high number of vaccinations that are given to the one-year-old cohort in developing countries [42]. Vaccination coverage was assumed to be 80% based on EPI data on the 4-year average coverage of measles vaccine in Cameroon between 2010 and 2013 [43], and more incidentally, the level of coverage assumed by a previous modelling paper on the potential long-term impact of a hypothetical schistosomiasis vaccine [44]. We based our estimate on the EPI data from Cameroon because: (a) it is a country with a high prevalence of onchocerciasis–loiasis co-endemicity, and therefore a potential beneficiary of an onchocerciasis vaccine, and (b) the demographic structure of EPIONCHO is based on data from this country [32]. We also varied the assumed level of coverage as part of our sensitivity analysis, choosing a more modest 60% coverage to reflect, perhaps, a lower degree of public confidence in a new vaccine compared to more familiar and established vaccines.

### Scenarios Explored and Model Output

We used the model to investigate (1) the beneficial impact of vaccination in terms of reductions in onchocerciasis infection and transmission in *O*. *volvulus–L*. *loa* co-endemic areas where ivermectin is contraindicated, and (2) the long-term dynamics of vaccine-induced protection against *O*. *volvulus* infection and how this may reduce the chance of infection recrudescence following elimination (and cessation of ivermectin MDA). We investigated these scenarios using three principal model outputs, all presented after 15 years of a hypothetical vaccination programme. These outputs are: (a) the mean microfilarial load in the human population as a whole and the age-stratified contribution to this mean; (b) the overall annual transmission potential (ATP, the average number of L3 larvae potentially received per person per year), and the age- and sex-specific contributions to the ATP; (c) the age-specific protection afforded by the vaccine against new infections.

**Fig 3:**
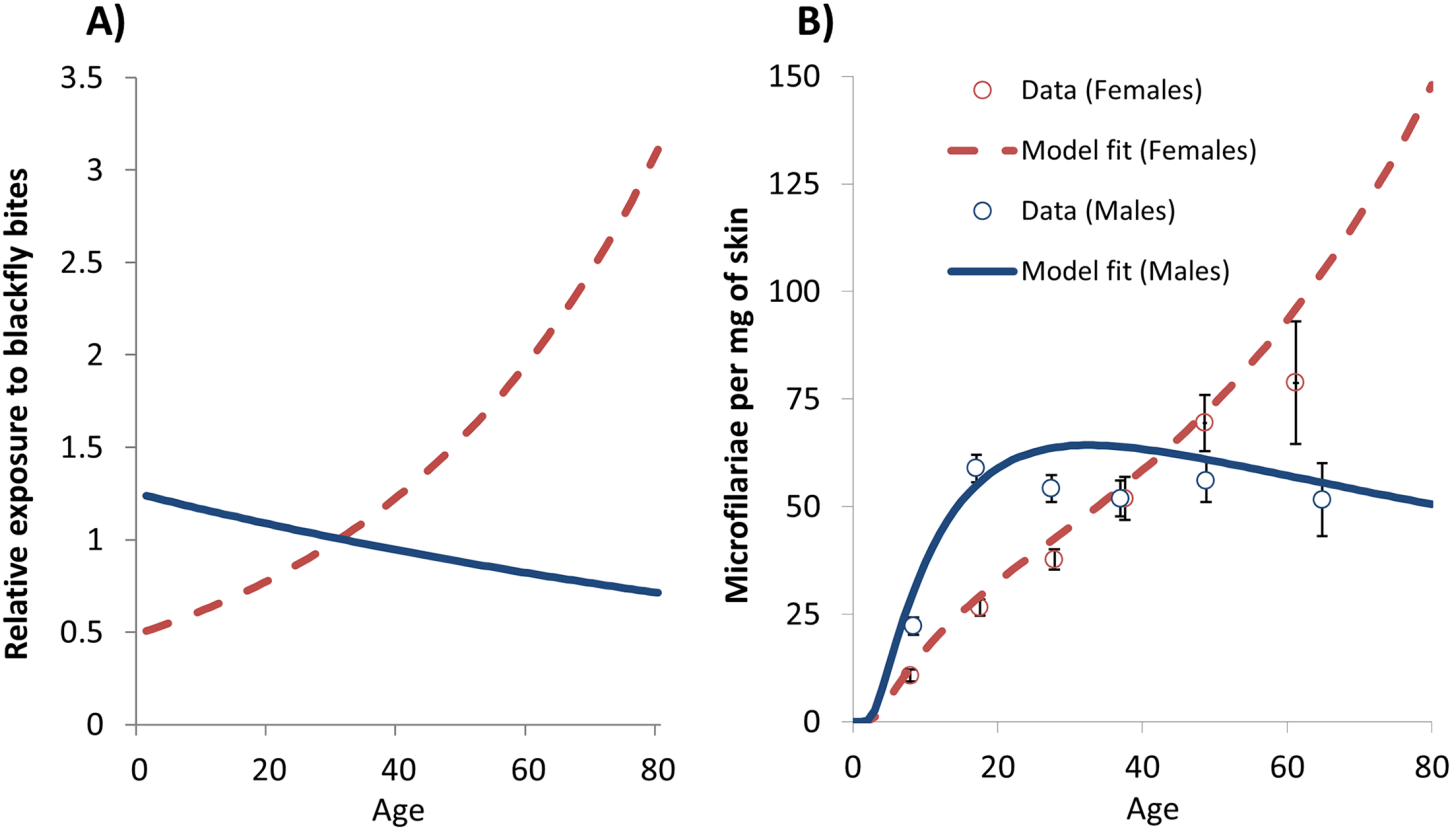
EPIONCHO’s underlying age- and sex-specific exposure and baseline microfilarial load profiles. **(A)** The age- and sex-specific exposure profiles to blackfly bites calibrated to reproduce the observed pre-control age-dependent microfilarial loads. **(B)** The age- and sex-specific microfilarial loads in African savannah settings of northern Cameroon [32]. Note that the fitting was performed using the individual data, not the binned data shown in Fig 2B. Note also that the legend on panel **(B)** applies to both panels **(A)** and **(B)**.

The age-stratified contribution to mean microfilarial load was obtained by multiplying the age- and sex-specific microfilarial loads (Fig 3B) times the proportion of the population within each corresponding demographic stratum (Fig 2A for age and Fig 2B for sex). The sum (grand total) of the age-stratified contribution yields the overall mean microfilarial load. The age- and sex-specific contribution to the ATP was calculated as the product of the following factors: i) the age- and sex-specific microfilarial loads; ii) the proportion of the population within each corresponding demographic stratum; iii) the proportion of blackfly bites taken on each demographic stratum (Fig 3A); iv) the annual biting rate (ABR); and v) the constraining density-dependent processes (parasite establishment and fly survival) acting on the development, to L3 larvae, of ingested mf within the blackfly [32]. The sum (grand total) of the age-and sex-stratified contribution to ATP yields the overall annual transmission potential.

## Results and Discussion

### Scenario 1: Onchocerciasis—Loiasis Co-endemic Areas

**Fig 4:**
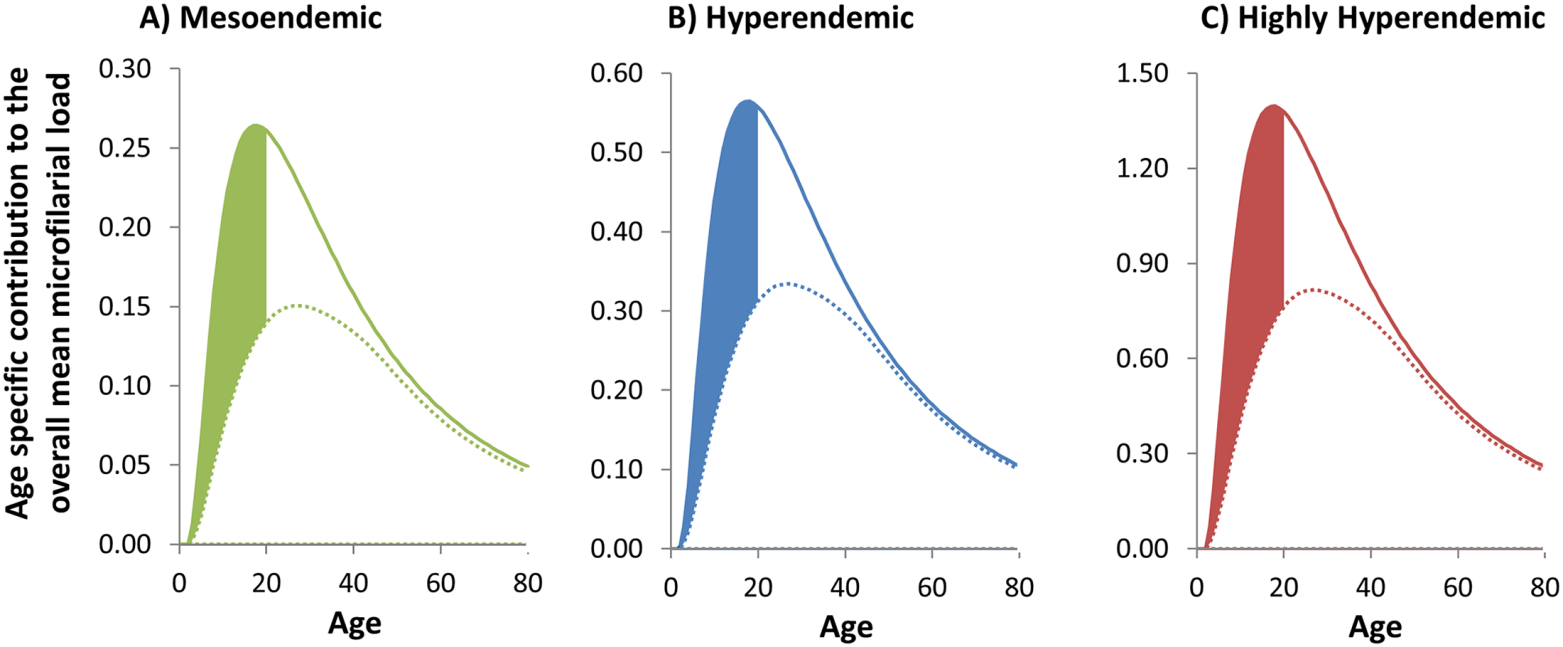
Long-term impact of vaccination on microfilarial load in the absence of ivermectin treatment. The green **(A)**, blue **(B)** and red **(C)** lines correspond to, respectively, a pre-control endemicity of 40%, 60%, and 80% microfilarial prevalence. The solid lines indicate the pre-control contribution of each group to the overall microfilarial load, which is the product of multiplying the microfilarial age- and sex specific profiles (Fig 3B) times the proportion of hosts in each demographic stratum, i.e. the proportion of hosts in each age and sex group (Fig 2). The sum total of the age- and sex-specific contributions yields the overall mean microfilarial load. The dotted lines correspond to the values after 15 years of vaccination. The shaded area illustrates the reduction in microfilarial load in those aged less than 20 years. Modelling assumptions are as follows: a vaccination programme targeting initially 1–5 year olds with continuous vaccination of one year olds after the first year of the programme; an initial prophylactic efficacy against the development of incoming worms of 50%; an initial therapeutic efficacy against skin microfilarial load of 90%; a mean duration of protective and therapeutic effects of 20 years (rate of decay = 0.05 per year) and an 80% coverage of vaccination.

Our modelling results indicate that the deployment of an onchocerciasis vaccine would have a substantial beneficial impact in *O*. *volvulus–L*. *loa* co-endemic areas where it may not be possible to deliver ivermectin MDA, or the population does not take treatment for fear of SAEs. However, these benefits take a considerable time to accrue since vaccinated individuals (one to five year olds initially and then only one year olds) need to age through the population into the more heavily exposed population age groups (Fig 3A). After 15 years of vaccination, the overall mean microfilarial load in the population is projected to decrease by 30% in highly hyper- and hyperendemic onchocerciasis foci and by 32% in mesoendemic foci (Table 2). Assuming a more modest 60% vaccination coverage (as opposed to the default 80%), the corresponding reductions are 23% (highly hyperendemic), 22% (hyperendemic) and 24% (mesoendemic) (S1 File, Table D). When the initial one- to five-year old ‘catch-up’ campaign is omitted and the programme comprises the continuous vaccination of five-year olds only (but see below for a discussion on caveats associated with this approach), the reductions in the highly hyper-, hyper- and mesoendemic foci, again after 15 years, are 24%, 24% and 26% respectively (S1 File, Table E). Fig 4 illustrates the profile of the age-specific contribution to overall mean microfilarial load, accounting for both demography of the population (Fig 2) and infection (Fig 3B). Although the reduction in the overall mean microfilarial load is somewhat modest compared to what could be achieved if it were possible to deliver ivermectin MDA [5], it is highly relevant that the most substantial reductions occur among younger members of the population. Previous studies have highlighted the crucial role played by exposure to heavy infection early in life on the risk of onchocerciasis-associated morbidity, blindness and excess mortality [36,45], and that for a given microfilarial load the relative risk of mortality is much greater in the <20 yr age group [43]. Hence, our modelling results suggest that an onchocerciasis vaccine would contribute to reduce the burden of disease and mortality in these populations, with most benefit afforded to those aged less than 20 years. In future, it will be important to determine whether a vaccine eliciting these anticipated reductions in onchocerciasis-associated disease and mortality could be delivered in a cost-effective manner. Like any intervention, this will crucially depend on the balance between the fixed and variable costs combined with the scale of the intervention (economies of scale) [46,47]. Currently, it is difficult to ascribe plausible costs to an onchocerciasis vaccination programme given the early stage of the vaccine’s development, and that no comparable vaccines or vaccination programmes exist for any other human helminthiasis. Besides, if ivermectin treatment were to be implemented in areas of onchocerciasis–loiasis co-endemicity with high risk of SAEs (those with a loiasis prevalence ≥ 20%), it would have to be on a test-and-treat basis in order to identify and exclude those with a high loiasis microfilaraemia and therefore most at risk, which would raise the costs over those of routine community-directed treatment with ivermectin (CDTI). In addition, measures would have to be put in place to monitor any SAEs that might occur and provide adequate care, and this would also elevate the costs of programmes based on ivermectin. These considerations would have to be taken into account in any cost-effectiveness comparison.

**Table 2:** Long-term impact of vaccination on onchocerciasis annual transmission potential and microfilarial load in the absence of ivermectin treatment under different assumptions of initial vaccine efficacy.

**A**	Years after vaccination				
	Pre-Control	5 years	10 years	15 years	30 years
Annual transmission potential (ATP) and percent reduction from baseline (%)					
Mesoendemic	88	76 (14%)	71 (19%)	69 (22%)	57 (35%)
Hyperendemic	373	324 (13%)	307 (18%)	294 (21%)	269 (28%)
Highly hyperendemic	4,365	3,778 (13%)	3,574 (18%)	3,419 (22%)	3,137 (28%)
Mean microfilarial load (arithmetic mean no. microfilariae/mg, all ages) and percent reduction (%)					
Mesoendemic	11.2	9.1 (19%)	8.3 (26%)	7.6 (32%)	6.3 (44%)
Hyperendemic	24.0	19.5 (19%)	18.0 (25%)	16.9 (30%)	15.2 (37%)
Highly hyperendemic	59.2	48.0 (19%)	44.3 (25%)	41.4 (30%)	36.7 (38%)
**B**					
Annual transmission potential (ATP) and percent reduction from baseline (%)					
Mesoendemic	88	75 (15%)	69 (22%)	64 (27%)	52 (41%)
Hyperendemic	373	319 (14%)	299 (20%)	283 (24%)	254 (32%)
Highly hyperendemic	4,365	3,723 (15%)	3,479 (20%)	3,291 (25%)	2,957 (32%)
Mean microfilarial load (arithmetic mean no. microfilariae/mg, all ages) and percent reduction (%)					
Mesoendemic	11.2	8.9 (21%)	8.0 (29%)	7.2 (36%)	5.7 (49%)
Hyperendemic	24.0	19.1 (20%)	17.3 (28%)	16.1 (33%)	13.9 (42%)
Highly hyperendemic	59.2	47.1 (20%)	42.6 (28%)	39.2 (34%)	33.7 (43%)

**A:** Model assumes an initial vaccine efficacy against the development of incoming worms of 50% and against skin microfilarial load of 90%. **B:** Model assumes a higher initial vaccine efficacy against the development of incoming worms of 70% and against skin microfilarial load of 95%. Results assume mean duration of prophylactic and therapeutic effects of 20 years (rate of decay = 0.05 per year) and an 80% coverage of vaccination. Annual transmission potential (ATP): the average number of L3 larvae potentially received per person per year.

**Fig 5:**
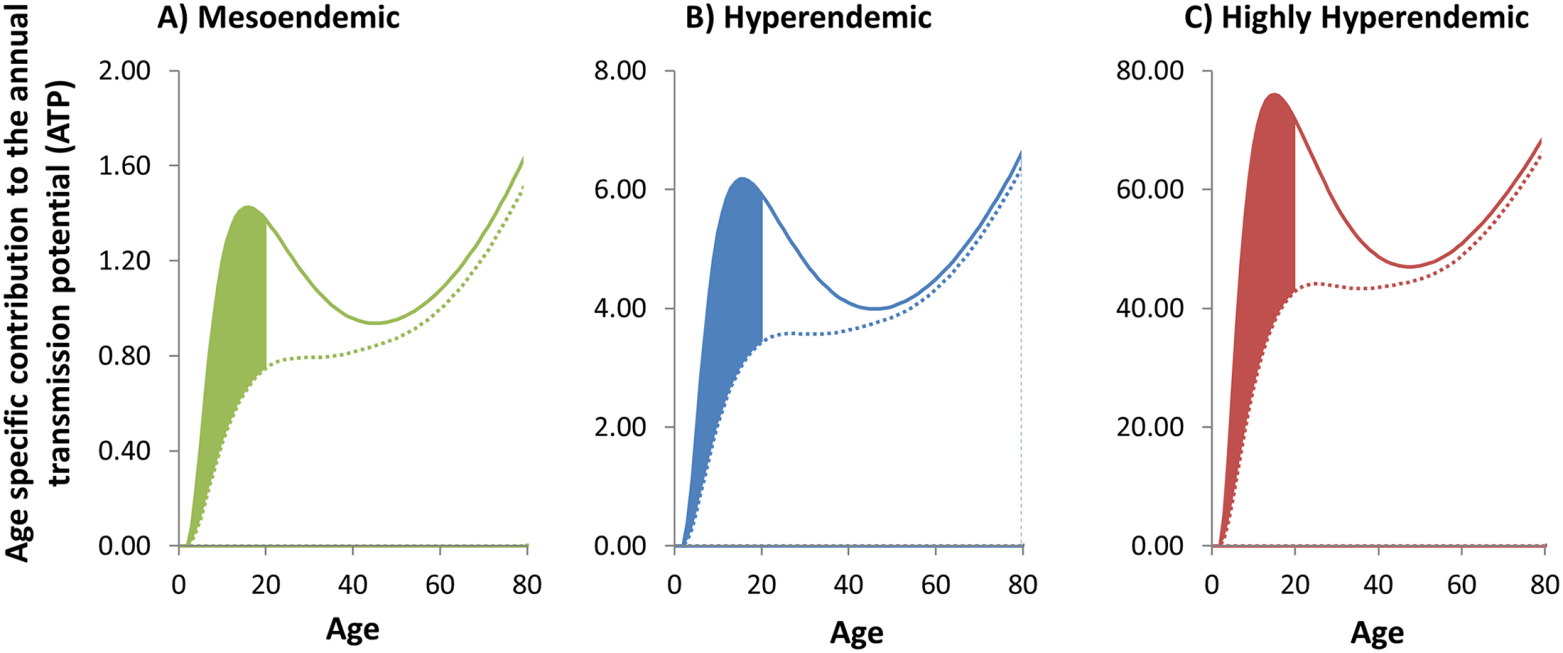
Long-term impact of vaccination on the overall contribution to onwards transmission by age groups in the host population in absence of ivermectin treatment. The green **(A)**, blue **(B)** and red **(C)** lines correspond to, respectively, a pre-control endemicity of 40%, 60%, and 80% microfilarial prevalence. The solid line indicates the baseline age-specific contribution to the annual transmission potential (ATP, no. L3/person/year). This is obtained as the product of multiplying the following factors: age- and sex-specific microfilarial loads; proportion of the population within each corresponding demographic stratum; proportion of blackfly bites taken on each demographic stratum (Fig 3A); annual biting rate; and the constraining (negative) density-dependent processes, acting on ingested microfilariae within the blackfly vector and on vector survival, that determine L3 output. The dotted lines correspond to the age-specific contributions to the ATP after 15 years of vaccination. The shaded area illustrates the reduction in contribution to transmission by those aged less than 20 years. Modelling assumptions on the initial vaccine efficacy and vaccine duration are as in Fig 4. The increasing contribution to ATP by older age groups is mainly due to women for whom microfilarial load and exposure to blackfly bites increases with age (Fig 3).

The ATP is projected to decrease by over 20% (Table 2), representing reductions in onchocerciasis transmission which would diminish the risk of *O*. *volvulus–L*. *loa* co-endemic areas acting as sources of infection to areas where treatment programmes are in the process of being scaled down or stopped. The reduction in the intensity of transmission (ATP), of 20%, is less than the reduction in the intensity of infection (microfilarial load), of 30%, because older women (particularly those aged ≥60 years), albeit comprising a relatively small proportion of the total population (Fig 2A), are both heavily exposed to biting blackflies (Fig 3A) and, at baseline, also heavily infected (Fig 3B). Like the age-specific contributions to the microfilarial load (Fig 4), the corresponding contributions to the ATP depicted in Fig 5 are most reduced in younger age groups, making these age groups, despite being most numerous in the population, the lowest contributors to infection transmission.

**Fig 6:**
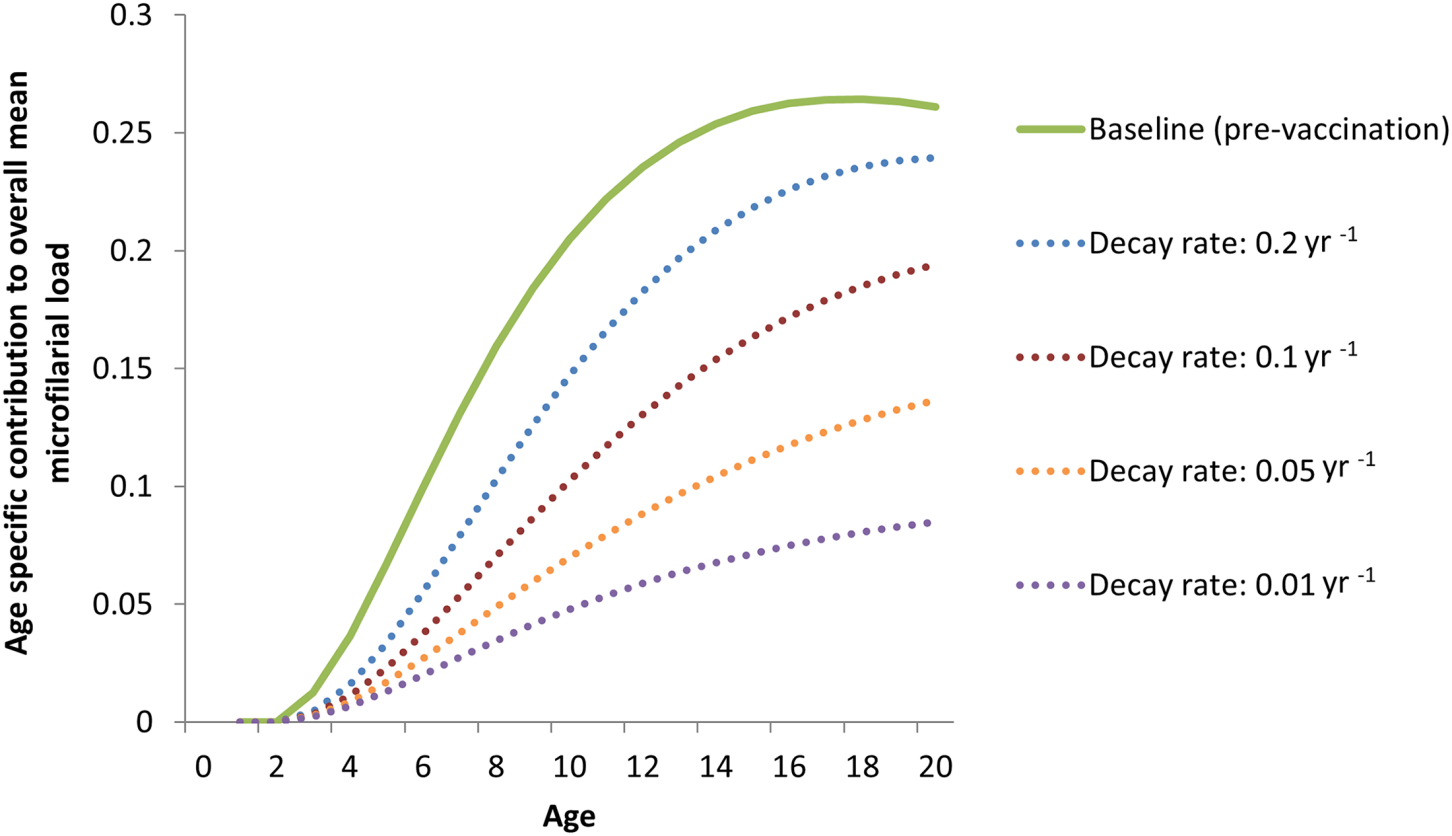
Sensitivity of the long-term reduction in microfilarial load in individuals under 20 years of age to the assumed rate of decay of vaccine efficacy. The mean duration of vaccine prophylactic (against incoming L3 larvae) and therapeutic (against microfilariae) activity is 1/the rate of decay (i.e. 5, 10, 20 and 50 years). We illustrate with a pre-control endemicity of 40% microfilarial prevalence. The solid line indicates the baseline (pre-control) contribution of each group to the overall microfilarial load, which is the product of multiplying the age- and sex-specific microfilarial loads (Fig 3B) times the proportion of the population within each corresponding demographic stratum (Fig 2). The dotted lines correspond to these contributions after 15 years of vaccination for decreasing waning rates of the prophylactic and therapeutic effects of the vaccine; the lower the rate, the greater the reduction in microfilarial loads achieved by the vaccination programme. Other assumptions as in Fig 4.

Reductions in microfilarial load and ATP are only marginally increased by assuming a greater initial vaccine efficacy; of 70% for prophylactic (against L3 larvae) efficacy, and of 95% for therapeutic (against mf) efficacy (Table 2B). By contrast, reductions in the assumed rate of waning of these vaccine effects have a marked impact on model outcomes (Fig 6). Therefore, it will be more important to invest in a vaccine with a slow rate of decay, effecting a long duration of protection—most likely mediated by the natural boosting effect of repeated infection challenges—than in an initially highly efficacious vaccine whose protective effects decay faster. This conclusion is in agreement with other modelling studies on potential schistosomiasis vaccines [44]. Therefore, our modelling helps to inform the most desirable properties of an onchocerciasis vaccine as an integral part of developing its target product profile (TPP).

Overall, the magnitude of the reduction in ATP elicited by an onchocerciasis vaccination programme would be unlikely to interrupt transmission *per se* and ultimately eliminate *O*. *volvulus* without concomitant and complementary interventions that can be safely and effectively implemented in areas of onchocerciasis–loiasis co-endemicity. Thus, in such areas of co-endemicity, it is envisaged that an onchocerciasis vaccine would represent an additional and complementary intervention strategy to be used in conjunction with interventions such as vector control or test and treat strategies using anti-*Wolbachia* macrofilaricidal drugs such as doxycycline [48], both of which are currently under consideration for recommendation as alternative treatment strategies (ATSs) by APOC.

## Scenario 2: Potential Influence on Infection Resurgence

**Fig 7:**
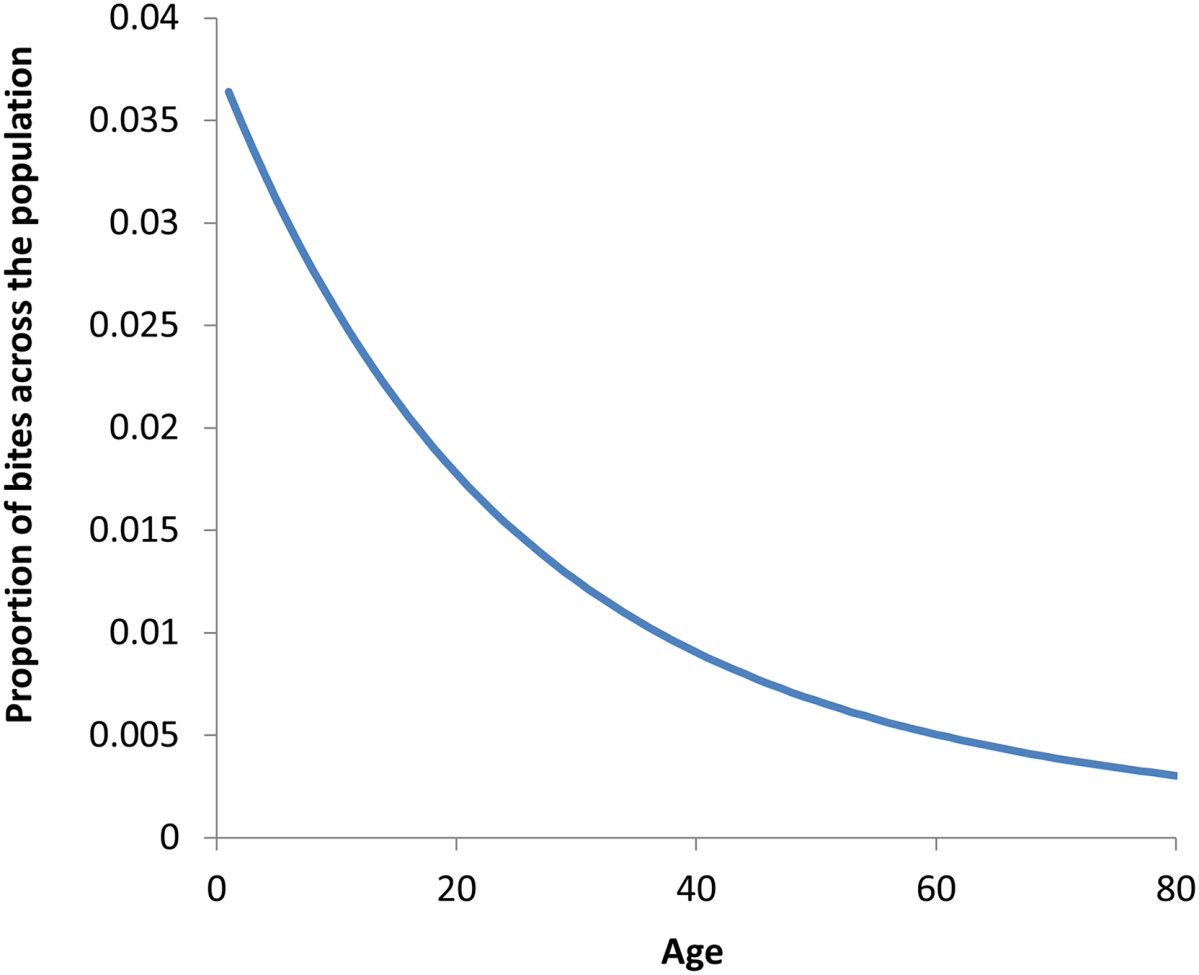
Model-predicted proportion of bites taken on each age group. The product of multiplying the age-and sex-specific exposure profiles to blackfly bites (Fig 3A) times the proportion of hosts in each age and sex group according to the demographic characteristics of the population (Fig 2).

**Fig 8:**
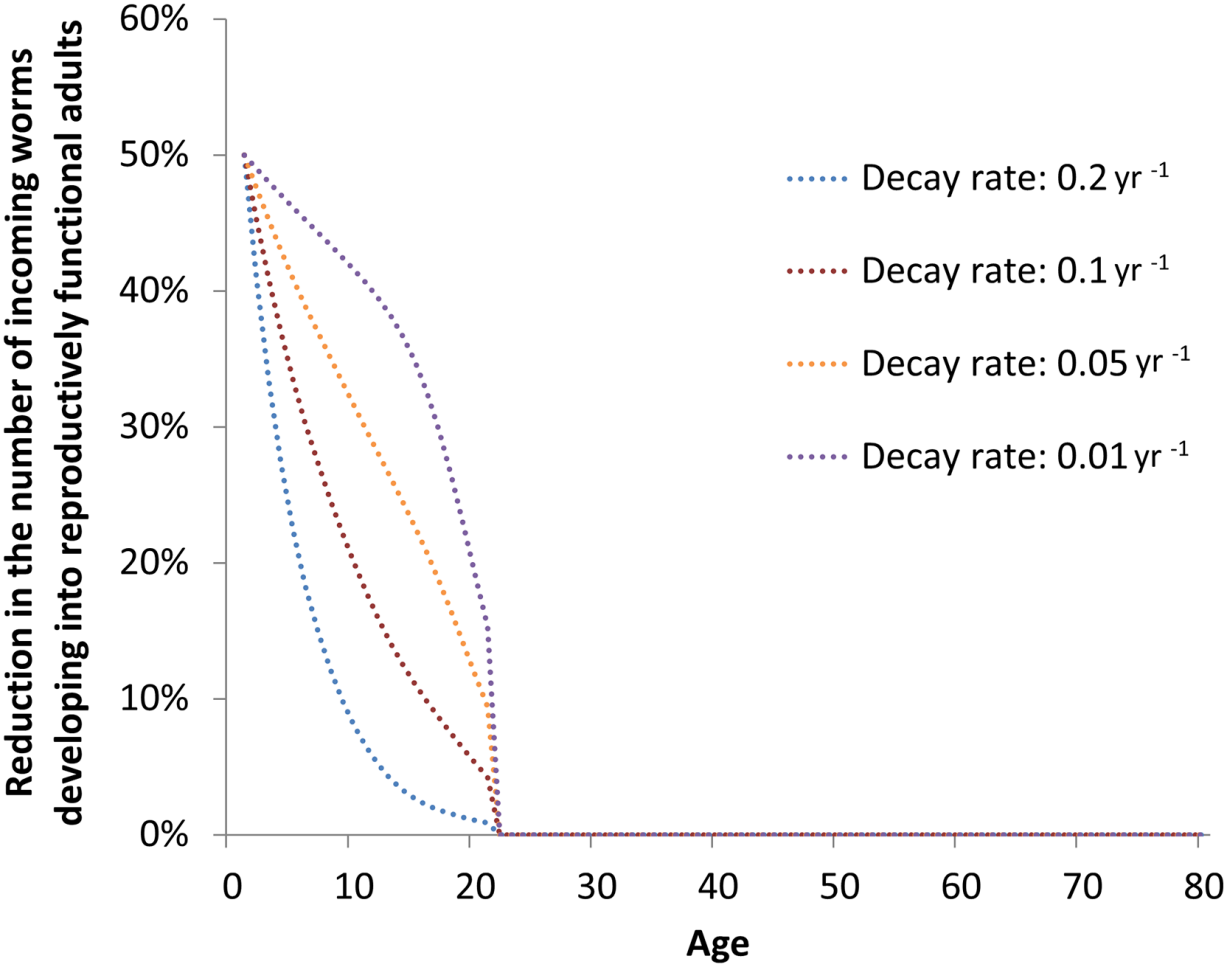
The profile of protection against incoming worms after 15 year of vaccination for different rates of decay of vaccine efficacy. The mean duration of vaccine prophylactic (against incoming L3 larvae) and therapeutic (against microfilariae) activity is 1/the decay rate (i.e. 5, 10, 20 and 50 years). The lower the waning rate, the greater the reduction in incoming worms achieved by the vaccination programme. Other assumptions as in Fig 4.

A key prerequisite to understanding how an onchocerciasis vaccine might mitigate the chances of reinfection from uncontrolled areas or areas with incomplete control is to consider the fraction of blackfly bites that are taken from different age groups by amalgamating the demographic structure of the population and the age- and sex-specific patterns of exposure to blackfly bites. This is illustrated in Fig 7 and demonstrates that the groups of the population protected by the vaccine (those aged less than 20 years) receive collectively most of the bites (because they are more numerous, Fig 2). Comparing this distribution (Fig 7) with the projected age-specific protection against incoming worms (prophylactic efficacy) after 15 year of vaccination (Fig 8), suggests that an onchocerciasis vaccine could markedly decrease the chance of onchocerciasis infection re-spreading to areas where treatment has been stopped (because it protects the age group who receive most bites). Hence, an onchocerciasis vaccine could help to protect the substantial investments already made by donors and stakeholders of ivermectin MDA programmes. However, this result is sensitive to the assumed rate of decay of vaccine protection (Fig 8), reinforcing the emphasis that should be placed in the TPP on achieving a vaccine with a long duration of protection.

The issues regarding recrudescence of infection in areas where ivermectin treatment has been stopped will have important programmatic implications, as having to recommence dismantled MDA campaigns is potentially expensive. Together, the Onchocerciasis Control Programme in West Africa (OCP, 1974–2002) and APOC (1995–2015) have cost over US$1 billion [49,50]—excluding economic costs (such as the donated ivermectin tablets and the time spent volunteered by community drug distributors [51]). The economic value of the donated ivermectin used in APOC (1995–2015) has been estimated to be US$3.9 billion (assuming 2.8 tablets per treatment and a commercial price of US$1.50 plus US$0.005 for shipping costs, per tablet) [50]. This highlights the important role a vaccine could have in protecting the substantial investments made by onchocerciasis control programmes, donors, and stakeholders in the global health community.

### Other Considerations and Research Needs

Based on elimination successes in Mali and Senegal [52,53], lessons learned when stopping onchocerciasis control in the OCP, and projections of the ONCHOSIM model [54], APOC has proposed provisional operational thresholds for treatment interruption followed by surveillance (pOTTIS). These comprise a microfilarial prevalence (by skin snipping) less than 5% in all surveyed villages and less than 1% in 90% of such villages, as well as less than 0.5 L3 larvae per 1,000 flies [55]. It is important to emphasize that these pOTTIS are not necessarily equivalent to transmission breakpoints, which represent a parasite density (and corresponding prevalence) below which the worm population would not be able to maintain itself due to the presence of Allee effects [11,56]. The magnitude of the transmission breakpoints is likely to be very locale-specific, depending on factors such as the parasite distribution and reproductive biology resulting from prolonged treatment, and the prevailing vector biting rates and competence for *O*. *volvulus* [57].

Although the pOTTIS have been validated in some foci—with low pre-control endemicity and highly seasonal transmission by savannah members of *S*. *damnosum s*.*l*. [52,53,58]—they will not necessarily hold in all epidemiological settings; particularly those with high pre-control endemicity, transmission rates and vector density. Furthermore, the current entomological threshold within these guidelines is measured per 1,000 flies and not per 1,000 parous flies (those which have previously fed on blood, laid eggs and survived gonotrophic cycle(s)). Consequently, it does not account for any potential differences in parity and survival rates among vector species in different seasons, or for different vector mixes when more than one simuliid species contributes to transmission in the same locale [59]. This, together with the poor sensitivity of skin snipping when infection levels are low [60], can lead to misleading conclusions regarding the level of ongoing transmission and potentially to treatment being stopped prematurely. An onchocerciasis vaccine would offer protection to populations after ivermectin distribution has ceased, and may reduce the chance of infection recrudescence in areas where treatment may have been stopped early. In addition, the use of an onchocerciasis vaccine would mitigate the consequences of a potential spread of ivermectin resistant *O*. *volvulus* [20,21].

It is also important to note that models such as EPIONCHO, ONCHOSIM, and others have been primarily calibrated with data collected in transmission areas of African savannah [32,57], an exception being the SIMON model [61], parameterised for a forest setting but not currently used for decision support in Africa. It is also important to note that models such as EPIONCHO, ONCHOSIM, and others have been primarily calibrated with data collected in transmission areas of African savannah [32,53], an exception being the SIMON model [57], parameterised for a forest setting but not currently used for decision support in Africa. Forest simuliid species do not exhibit the same degrees of density-dependent constraint on the fraction of incoming microfilariae that successfully establish in the thoracic muscles of the flies [57], resulting in higher numbers of L3 larvae per forest fly [62] and corresponding transmission potentials [63]. This could mean that forest blackflies are more efficient transmitters of infection, although little is known about other density dependencies that might mitigate this effect, such as the degree of density-dependent excess mortality inflicted on infected blackflies. Parasitological data on infection intensity (microfilarial loads) combined with entomological data on annual biting rates collected from communities in forest settings could help to infer vector efficiency indirectly, yet such data are somewhat scarce. It remains an important research need to parameterise onchocerciasis models to reflect the epidemiology and transmission of forest onchocerciasis, as it is in these areas that onchocerciasis–loiasis co-endemicity represents a barrier to elimination [14], and the use of an onchocerciasis vaccine would be highly desirable as one of a number of complementary interventions forming a multipronged strategy.

Other modelling studies have been conducted to explore the epidemiological impact of helminth vaccines such as for human [40,44] and zoonotic [64] schistosomiasis, and hookworm infection [65]. In particular the latter also explored vaccination of older age groups (school-age children). We refrained from doing this because it has been shown that helminth vaccines may not be efficacious in hosts who are already infected due to the immunomodulatory effects of helminth infection [66]. However, in areas of intense onchocerciasis transmission where ivermectin has not yet been deployed, the under 5-year olds may be patently infected and the 1-year olds pre-patently infected. These challenges will have to be taken into account when optimising the design of the vaccines and vaccination programmes. Since skin and blood samples are seldom taken from these age groups during onchocerciasis surveys, data to inform the (immuno-)epidemiology of the infection in young children are scarce (but see [67,68]). The development of *O*. *volvulus*-specific biomarkers for detection of active infection is a pressing research need [69,70].

A potential caveat of the vaccination strategy discussed in this paper would be the possibility of SAEs was there cross-reactivity between *O*. *volvulus* and *L*. *loa* with respect to the therapeutic effect of the vaccine against microfilariae. However, the amino acid identity between the three candidate *O*. *volvulus* proteins and their counterparts in *L*. *loa* amount only at 52% for *Ov*-RAL-2, 58% for *Ov*-CPI-2M and 71% for *Ov*-103, and therefore it is unlikely that there would be substantial cross efficacy at immunologically-mediated killing of microfilariae. Notwithstanding this seemingly low risk, this issue has not yet been tested in animal models of loiasis, but experimental models are being developed [71] that would allow investigation of this question if a patent infection could be established. More recently, a newly developed co-infection model of *O*. *ochengi* and *L*. *loa* microfilariae in Mongolian jirds (*Meriones unguiculatus*) has been established at the University of Buea, Cameroon, by Dr. Fidelis Cho-Ngwa (personal communication). This immunocompetent jird model was developed for the simultaneous testing of potential macrofilaricides on *O*. *ochengi* and *L*. *loa* microfilariae in the same animal. This counter screen is important in confirming that a drug, whilst killing adult worms *in vitro* or *in vivo*, will not kill *L*. *loa* microfilariae in a host with a fully intact immune system (as occurs in co-infected humans). This model could be also used to investigate the question of immunological cross reactivity (the similarity between *O*. *volvulus* and *O*. *ochengi* for all three proteins mentioned above is ≥99%), by immunizing with the recombinant antigens and then challenging with *O*. *ochengi* and *L*. *loa* microfilariae, following their mortality and any ensuing pathology.

Developing quantitative tools that allow rigorous exploration of the considerations described above will be essential for assessing the true cost-effectiveness of onchocerciasis vaccination. In particular, this work highlights the importance of developing spatially-explicit transmission models with which to investigate and quantify the probability of infection being re-introduced in successfully controlled areas from others with ongoing transmission. The results of the analysis clearly show the importance of obtaining reliable estimates of the duration of vaccine protection, i.e. the reciprocal of the rate at which vaccine efficacy would decay. This property of the vaccine will be more important than initial vaccine efficacy in terms of the long-term impact of vaccination campaigns

## Supporting Information

S1 File Description of the EPIONCHO model, equations, calibration, modifications to incorporate vaccination and model vaccine efficacy, and results under varying assumptions of coverage and age groups targeted. Text A: Model Description. Text B: Model Equations. Text C: Modelling Vaccine Efficacy. Table A: Summary of baseline (pre-control) modelled epidemiological scenarios. Table B: Definition and values of parameters and variables for the onchocerciasis population dynamics model EPIONCHO. Table C: Definition and values of parameters for mating probability and microfilarial prevalence calculations. Table D: Long-term impact of vaccination on onchocerciasis annual transmission potential and microfilarial load in the absence of ivermectin treatment under different assumptions of the vaccine coverage. Table E: Long-term impact of vaccination on onchocerciasis annual transmission potential and microfilarial load in the absence of ivermectin treatment under different assumptions of the targeted age group.
(DOCX)

## References

[1] World Health Organization. Accelerating work to overcome the global impact of neglected tropical diseases—A roadmap for implementation. Geneva: WHO. 2012. http://www.who.int/neglected_diseases/NTD_RoadMap_2012_Fullversion.pdf; accessed 17th June 2015.

[2] London Declaration on Neglected Tropical Diseases. Ending the neglect and reaching 2020 goals. London, UK. 2012. http://unitingtocombatntds.org/downloads/press/ntd_event_london_declaration_on_ntds.pdf; accessed 13th May 2015.

[3] World Health Organization. 18th Session of the Joint Action Forum. Bujumbura, Burundi. 2012. http://www.who.int/apoc/media/Journal_du_FAC_day_4_Anglais.pdf; accessed 13th May 2015.

[4] Bill & Melinda Gates Foundation. Gates Annual letter Our Big Bet for the Future. 2015. www.gatesletter.com; accessed 13th May 2015.

[5] Turner HC, Walker M, Churcher TS, Osei-Atweneboana MY, Biritwum N-K, Hopkins A, et al Reaching the London Declaration on Neglected Tropical Diseases goals for onchocerciasis: an economic evaluation of increasing the frequency of ivermectin treatment in Africa. Clin Infect Dis. 2014;59(7): 923–932. 10.1093/cid/ciu467 24944228 PMC4166981

[6] Turner HC, Walker M, Attah SK, Opoku NO, Awadzi K, Kuesel AC, et al The potential impact of moxidectin on onchocerciasis elimination in Africa: an economic evaluation based on the Phase II clinical trial data. Parasit Vectors. 2015;8: 167 10.1186/s13071-015-0779-4 25889256 PMC4381491

[7] Turner HC, Walker M, Churcher TS, Basáñez MG. Modelling the impact of ivermectin on River Blindness and its burden of morbidity and mortality in African savannah: EpiOncho projections. Parasit Vectors. 2014;7: 241 10.1186/1756-3305-7-241 24886747 PMC4037555

[8] Turner HC, Churcher TS, Walker M, Osei-Atweneboana MY, Prichard RK, Basáñez MG. Uncertainty surrounding projections of the long-term impact of ivermectin treatment for human onchocerciasis. PLoS Negl Trop Dis. 2013;7(4):: e2169 10.1371/journal.pntd.0002169 23634234 PMC3636241

[9] Winnen M, Plaisier AP, Alley ES, Nagelkerke NJD, van Oortmarssen G, Boatin BA, et al Can ivermectin mass treatments eliminate onchocerciasis in Africa? Bull World Health Organ. 2002;80(5): 384–391. 12077614 PMC2567795

[10] Coffeng LE, Stolk WA, Hoerauf A, Habbema D, Bakker R, Hopkins AD, et al Elimination of African onchocerciasis: modeling the impact of increasing the frequency of ivermectin mass treatment. PloS One. 2014;9(12): e115886 10.1371/journal.pone.0115886 25545677 PMC4278850

[11] Duerr HP, Raddatz G, Eichner M. Control of onchocerciasis in Africa: threshold shifts, breakpoints and rules for elimination. Int J Parasitol. 2011;41(5): 581–589. 10.1016/j.ijpara.2010.12.009 21255577

[12] Prichard RK, Basáñez MG, Boatin BA, McCarthy JS, Garcia HH, Yang GJ, et al A research agenda for helminth diseases of humans: intervention for control and elimination. PLoS Negl Trop Dis. 2012;6(4): e1549 10.1371/journal.pntd.0001549 22545163 PMC3335868

[13] Hotez PJ, Bottazzi ME, Zhan B, Makepeace BL, Klei TR, Abraham D, et al The Onchocerciasis Vaccine for Africa—TOVA—Initiative. PLoS Negl Trop Dis. 2015;9(1): e0003422 10.1371/journal.pntd.0003422 25634641 PMC4310604

[14] Zouré HGM, Wanji S, Noma M, Amazigo UV, Diggle PJ, Tekle AH, et al The geographic distribution of *Loa loa* in Africa: results of large-scale implementation of the Rapid Assessment Procedure for Loiasis (RAPLOA). PLoS Negl Trop Dis. 2011;5(6): e1210 10.1371/journal.pntd.0001210 21738809 PMC3125145

[15] Thomson MC, Obsomer V, Dunne M, Connor SJ, Molyneux DH. Satellite mapping of *Loa loa* prevalence in relation to ivermectin use in west and central Africa. Lancet. 2000;356(9235): 1077–1078. 11009145 10.1016/s0140-6736(00)02733-1

[16] Boussinesq M, Gardon J, Kamgno J, Pion SDS, Gardon-Wendel N, Chippaux JP. Relationships between the prevalence and intensity of *Loa loa* infection in the Central province of Cameroon. Ann Trop Med Parasitol. 2001;95(5): 495–507. 11487371 10.1080/00034980120073184

[17] Kelly-Hope LA, Cano J, Stanton MC, Bockarie MJ, Molyneux DH. Innovative tools for assessing risks for severe adverse events in areas of overlapping *Loa loa* and other filarial distributions: the application of micro-stratification mapping. Parasit Vectors. 2014;7: 307 10.1186/1756-3305-7-307 24992829 PMC4101798

[18] Awadzi K, Attah SK, Addy ET, Opoku NO, Quartey BT, Lazdins-Helds JK, et al Thirty-month follow-up of sub-optimal responders to multiple treatments with ivermectin, in two onchocerciasis-endemic foci in Ghana. Ann Trop Med Parasitol. 2004;98(4): 359–370. 15228717 10.1179/000349804225003442

[19] Awadzi K, Boakye DA, Edwards G, Opoku NO, Attah SK, Osei-Atweneboana MY, et al An investigation of persistent microfilaridermias despite multiple treatments with ivermectin, in two onchocerciasis-endemic foci in Ghana. Ann Trop Med Parasitol. 2004;98(3): 231–249. 15119969 10.1179/000349804225003253

[20] Osei-Atweneboana MY, Eng JKL, Boakye DA, Gyapong JO, Prichard RK. Prevalence and intensity of *Onchocerca volvulus* infection and efficacy of ivermectin in endemic communities in Ghana: a two-phase epidemiological study. Lancet. 2007;369 (9578): 2021–2029. 17574093 10.1016/S0140-6736(07)60942-8

[21] Churcher TS, Pion SDS, Osei-Atweneboana MY, Prichard RK, Awadzi K, Boussinesq M, et al Identifying sub-optimal responses to ivermectin in the treatment of River Blindness. Proc Natl Acad Sci U S A. 2009;106 (39): 16716–16721. 10.1073/pnas.0906176106 19805362 PMC2757820

[22] The onchocerciasis vaccine for Africa (TOVA) initiative. 2015. http://www.riverblindnessvaccinetova.org/; accessed 13th May 2015. 10.1371/journal.pntd.0003422 PMC4310604 25634641

[23] The Onchocerciasis Vaccine for Africa (TOVA) Initiative prospectus. 2015. http://www.riverblindnessvaccinetova.org/sites/default/files/atoms/files/River_Blindness_Prospectus%20Final.pdf; accessed 13th May 2015. 10.1371/journal.pntd.0003422 PMC4310604 25634641

[24] Cook JA, Steel C, Ottesen EA. Towards a vaccine for onchocerciasis. Trends Parasitol. 2001;17(12): 555–558. 11756017 10.1016/s1471-4922(01)02115-8

[25] Lustigman S, James ER, Tawe W, Abraham D. Towards a recombinant antigen vaccine against *Onchocerca volvulus* . Trends Parasitol. 2002;18(3): 135–141. 11854092 10.1016/s1471-4922(01)02211-5

[26] Hess JA, Zhan B, Bonne-Année S, Deckman JM, Bottazzi ME, Hotez PJ, et al Vaccines to combat river blindness: expression, selection and formulation of vaccines against infection with *Onchocerca volvulus* in a mouse model. Int J Parasitol. 2014;44(9): 637–646. 10.1016/j.ijpara.2014.04.006 24907553 PMC4118642

[27] Babayan SA, Allen JE, Taylor DW. Future prospects and challenges of vaccines against filariasis. Parasite Immunol. 2012;34(5): 243–253. 10.1111/j.1365-3024.2011.01350.x 22150082

[28] Arumugam S, Wei J, Ward D, Abraham D, Lustigman S, Zhan B, et al Vaccination with a genetically modified *Brugia malayi* cysteine protease inhibitor-2 reduces adult parasite numbers and affects the fertility of female worms following a subcutaneous challenge of Mongolian gerbils (*Meriones unguiculatus*) with *B*. *malayi* infective larvae. Int J Parasitol. 2014;44(10): 675–679. 10.1016/j.ijpara.2014.05.003 24929131 PMC5335902

[29] Babayan SA, Luo H, Gray N, Taylor DW, Allen JE. Deletion of parasite immune modulatory sequences combined with immune activating signals enhances vaccine mediated protection against filarial nematodes. PLoS Negl Trop Dis. 2012;6(12): e1968 10.1371/journal.pntd.0001968 23301106 PMC3531514

[30] Ziewer S, Hübner MP, Dubben B, Hoffmann WH, Bain O, Martin C, et al Immunization with *L*. *sigmodontis* microfilariae reduces peripheral microfilaraemia after challenge infection by inhibition of filarial embryogenesis. PLoS Negl Trop Dis. 2012;6(3): e1558 10.1371/journal.pntd.0001558 22413031 PMC3295809

[31] Basáñez MG, Boussinesq M. Population biology of human onchocerciasis. Philos Trans R Soc Lond B Biol Sci. 1999;354(1384): 809–826. 10365406 10.1098/rstb.1999.0433 PMC1692549

[32] Filipe JAN, Boussinesq M, Renz A, Collins RC, Vivas-Martinez S, Grillet ME et al Human infection patterns and heterogeneous exposure in river blindness. Proc Natl Acad Sci U S A. 2005;102(42): 15265–15270. 16217028 10.1073/pnas.0502659102 PMC1257694

[33] Basáñez MG, Pion SDS, Boakes E, Filipe JAN, Churcher TS, Boussinesq M. Effect of single-dose ivermectin on *Onchocerca volvulus*: a systematic review and meta-analysis. Lancet Infect Dis. 2008;8(5): 310–322. 10.1016/S1473-3099(08)70099-9 18471776

[34] Churcher TS, Basáñez MG. Density dependence and the spread of anthelmintic resistance. Evolution. 2008;62(3): 528–537. 17983465 10.1111/j.1558-5646.2007.00290.x

[35] Anderson J, Fuglsang H, Hamilton PJS, de Marshall TF. Studies on onchocerciasis in the United Cameroon Republic II. Comparison of onchocerciasis in rain-forest and Sudan-savanna. Trans R Soc Trop Med Hyg. 1974;68(3): 209–222. 4421167 10.1016/0035-9203(74)90117-5

[36] Renz A, Fuglsang H, Anderson J. Studies on the dynamics of transmission of onchocerciasis in a Sudan-savanna area of North Cameroon IV. The different exposure to *Simulium* bites and transmission of boys and girls and men and women, and the resulting manifestations of onchocerciasis. Ann Trop Med Parasitol. 1987; 81(3): 253–262. 3662667 10.1080/00034983.1987.11812118

[37] Bradley JE, Whitworth J, Basáñez MG. Onchocerciasis In: Wakelin D, Cox F, Despommier D, Gillespie S, editors. Topley and Wilson’s Microbiology and Microbial Infections. 10th ed London: London: Hodder Arnold; 2005 pp. 781–801.

[38] Duke BOL (1990) Human onchocerciasis—an overview of the disease. Acta Leiden 59(1–2): 9–24. 2198761

[39] Remme JHF, Ba O, Dadzie KY, Karam M (1986) A force-of-infection model for onchocerciasis and its applications in the epidemiological evaluation of the Onchocerciasis Control Programme in the Volta River basin area. Bull World Health Organ 64: 667–681. 3492300 PMC2490951

[40] Chan MS, Woolhouse ME, Bundy DAP (1997) Human schistosomiasis: potential long-term consequences of vaccination programmes. Vaccine 15: 1545–1550. 9330466 10.1016/s0264-410x(97)00071-6

[41] Keja K, Chan C, Hayden G, Henderson RH (1988) Expanded programme on immunization. World Health Stat Q 41: 59–63. 3176515

[42] World Health Organization. WHO recommendations for routine immunization—summary tables. 2015. http://www.who.int/immunization/policy/immunization_tables/en/; accessed 13th May 2015.

[43] The World Bank. Immunization, measles (% of children ages 12–23 months). 2013. http://data.worldbank.org/indicator/SH.IMM.MEAS?order=wbapi_data_value_2011+wbapi_data_value+w; accessed 13th May 2015.

[44] Woolhouse ME. Human schistosomiasis: potential consequences of vaccination. Vaccine. 1995;13(12): 1045–1050. 7491810 10.1016/0264-410x(95)00083-d

[45] Walker M, Little MP, Wagner KS, Soumbey-Alley EW, Boatin BA, Basáñez MG. Density-dependent mortality of the human host in onchocerciasis: relationships between microfilarial load and excess mortality. PLoS Negl Trop Dis. 2012;6(3): e1578 10.1371/journal.pntd.0001578 22479660 PMC3313942

[46] Turner HC, Walker M, French MD, Blake IM, Churcher TS, Basáñez MG. Neglected tools for neglected diseases: mathematical models in economic evaluations. Trends Parasitol. 2014;30(12): 562–570. 10.1016/j.pt.2014.10.001 25455565

[47] Turner HC, Truscott JE, Hollingsworth TD, Bettis AA, Brooker SJ, Anderson RM. Cost and cost-effectiveness of soil-transmitted helminth treatment programmes: systematic review and research needs. Parasit Vectors. 2015; In press. 10.1186/s13071-015-0885-3 PMC4499443 26137945

[48] Walker M, Specht S, Churcher TS, Hoerauf A, Taylor MJ, Basáñez MG. Therapeutic efficacy and macrofilaricidal activity of doxycycline for the treatment of River Blindness. Clin Infect Dis. 2015;60(8): 1199–1207. 10.1093/cid/ciu1152 25537873 PMC4370165

[49] Remme JHF, Feenstra P, Lever PR, Medici AC, Morel CM. Tropical diseases targeted for elimination: chagas disease, lymphatic dilariasis, onchocerciasis, and leprosy In: Jamison DT, Breman JG, Measham AR, editors. Disease Control Priorities in Developing Countries. New York: Oxford University Press; 2006 pp. 433–449. 21250324

[50] Coffeng LE, Stolk WA, Zouré HGM, Veerman JL, Agblewonu KB, Murdoch ME, et al African Programme for Onchocerciasis Control 1995–2015: model-estimated health impact and cost. PLoS Negl Trop Dis. 2013;7(1): e2032 10.1371/journal.pntd.0002032 23383355 PMC3561133

[51] Turner HC, Osei-Atweneboana MY, Walker M, Tettevi EJ, Churcher TS, Asiedu O, et al The cost of annual versus biannual community-directed treatment with ivermectin: Ghana as a case study PLoS Negl Trop Dis. 2013;7(9): e2452 10.1371/journal.pntd.0002452 24069497 PMC3777881

[52] Diawara L, Traoré MO, Badji A, Bissan Y, Doumbia K, Goita SF, et al Feasibility of onchocerciasis elimination with ivermectin treatment in endemic foci in Africa: first evidence from studies in Mali and Senegal. PLoS Negl Trop Dis. 2009;3(7): e497 10.1371/journal.pntd.0000497 19621091 PMC2710500

[53] Tekle A, Elhassan E, Isiyaku S, Amazigo U, Bush S, Noma M, et al Impact of long-term treatment of onchocerciasis with ivermectin in Kaduna State, Nigeria: first evidence of the potential for elimination in the operational area of the African Programme for Onchocerciasis Control. Parasit Vectors. 2012;5: 28 10.1186/1756-3305-5-28 22313631 PMC3296569

[54] Plaisier AP, van Oortmarssen GJ, Habbema JDF, Remme JHF, Alley ES. ONCHOSIM: a model and computer simulation program for the transmission and control of onchocerciasis. Comput Methods Programs Biomed. 1990;31(1): 43–56. 2311368 10.1016/0169-2607(90)90030-d

[55] African Programme for Onchocerciasis Control. Conceptual and operational framework of onchocerciasis elimination with ivermectin treatment. WHO/APOC. 2010. http://www.who.int/apoc/oncho_elimination_report_english.pdf; accessed 13th May 2015.

[56] Anderson RM, May RM. Infectious diseases of humans Dynamics and control. Oxford: Oxford Science Publications; 1992.

[57] Basáñez MG, Churcher TS, Grillet ME. *Onchocerca-Simulium* interactions and the population and evolutionary biology of *Onchocerca volvulus* . Adv Parasitol. 2009;68: 263–313. 10.1016/S0065-308X(08)00611-8 19289198

[58] Traoré MO, Sarr MD, Badji A, Bissan Y, Diawara L, Doumbia K, et al Proof-of-principle of onchocerciasis elimination with ivermectin treatment in endemic foci in Africa: final results of a study in Mali and Senegal. PLoS Negl Trop Dis. 2012;6: e1825 10.1371/journal.pntd.0001825 23029586 PMC3441490

[59] Lamberton PHL, Cheke RA, Walker M, Winskill P, Osei-Atweneboana MY, Tirados I, et al Onchocerciasis transmission in Ghana: biting and parous rates of host-seeking sibling species of the *Simulium damnosum* complex. Parasit Vectors. 2014;7: 511 10.1186/s13071-014-0511-9 25413569 PMC4247625

[60] McCarthy JS, Lustigman S, Yang G-J, Barakat RM, García HH, Sripa B, et al A research agenda for helminth diseases of humans: diagnostics for control and elimination programmes. PLoS Negl Trop Dis. 2012;6(4): e1601 10.1371/journal.pntd.0001601 22545166 PMC3335877

[61] Davies JB. Description of a computer model of forest onchocerciasis transmission and its application to field scenarios of vector control and chemotherapy. Ann Trop Med Parasitol. 1993;87(1): 41–63. 8346991 10.1080/00034983.1993.11812738

[62] Cheke RA, Garms R. Indices of onchocerciasis transmission by different members of the *Simulium damnosum* complex conflict with the paradigm of forest and savanna parasite strains. Acta Trop. 2013;125(1): 43–52. 10.1016/j.actatropica.2012.09.002 22995985

[63] Duke BOL, Moore PJ, Anderson J. Studies on factors influencing the transmission of onchocerciasis. VII. A comparison of the *Onchocerca volvulus* transmission potentials of *Simulium damnosum* populations in four Cameroon rain-forest villages and the pattern of onchocerciasis associated therewith. Ann Trop Med Parasitol. 1972;66(2): 219–234. 5038247

[64] Williams GM, Sleigh AC, Li Y, Feng Z, Davis GM, Chen H, et al Mathematical modelling of *schistosomiasis japonica*: comparison of control strategies in the People's Republic of China. Acta Trop. 2002; 82(2): 253–262. 12020899 10.1016/s0001-706x(02)00017-7

[65] Sabatelli L, Ghani AC, Rodrigues LC, Hotez PJ, Brooker S. Modelling heterogeneity and the impact of chemotherapy and vaccination against human hookworm. J R Soc Interface. 2008;5(28): 1329–1341. 10.1098/rsif.2007.1255 18331978 PMC2607437

[66] Hewitson JP, Maizels RM. Vaccination against helminth parasite infections. Expert Rev Vaccines. 2014;13(4): 473–487. 24606541 10.1586/14760584.2014.893195

[67] Kirch AK, Duerr HP, Boatin B, Alley WS, Hoffmann WH, Schulz-Key H, et al Impact of parental onchocerciasis and intensity of transmission on development and persistence of *Onchocerca volvulus* infection in offspring: an 18 year follow-up study. Parasitology. 2003; 127(4): 327–335. 14636019 10.1017/s0031182003003834

[68] Prost A, Gorim de Ponsay E. [The epidemiological significance of neo-natal parasitism with microfilariae of *Onchocerca volvulus* (author's transl)]. Tropenmed Parasitol. 1979;30(4): 477–481. [Article in French]. 575449

[69] Globisch D, Moreno AY, Hixon MS, Nunes AA, Denery JR, Specht S, et al *Onchocerca volvulus*-neurotransmitter tyramine is a biomarker for river blindness. Proc Natl Acad Sci U S A. 2013;110(11): 4218–4223. 10.1073/pnas.1221969110 23440222 PMC3600455

[70] Quintana JF, Makepeace BL, Babayan SA, Ivens A, Pfarr KM, Blaxter M et al Extracellular *Onchocerca*-derived small RNAs in host nodules and blood. Parasit Vectors. 2015;8: 58 10.1186/s13071-015-0656-1 25623184 PMC4316651

[71] Tendongfor N, Wanji S, Ngwa JC, Esum ME, Specht S, Enyong P, et al The human parasite *Loa loa* in cytokine and cytokine receptor gene knock out BALB/c mice: survival, development and localization. Parasit Vectors. 2012;5: 43 10.1186/1756-3305-5-43 22348321 PMC3305519

[72] Prost A, Hervouet JP, Thylefors B. [Epidemiologic status of onchocerciasis]. Bull World Health Organ. 1979;57(4): 655–662. [Article in French]. 316743 PMC2395839

