# Supplementary material for: Human Onchocerciasis: Modelling the Potential Long-term Consequences of a Vaccination Programme
Source: PLoS Negl Trop Dis. 2015 Jul 17;9(7):e0003938. doi: 10.1371/journal.pntd.0003938 (PMC4506122; doi:10.1371/journal.pntd.0003938)
Supplement: S1 File — Text A: Model Description. Text B: Model Equations. Text C: Modelling Vaccine Efficacy. Table A: Summary of baseline (pre-control) modelled epidemiological scenarios. Table B: Definition and values of parameters and variables for the onchocerciasis population dynamics model EPIONCHO. Table C: Definition and values of parameters for mating probability and microfilarial prevalence calculations. Table D: Long-term impact of vaccination on onchocerciasis annual transmission potential and microfilarial load in the absence of ivermectin treatment under different assumptions of the vaccine coverage. Table E: Long-term impact of vaccination on onchocerciasis annual transmission potential and microfilarial load in the absence of ivermectin treatment under different assumptions of the targeted age group. (DOCX) [file pntd.0003938.s001.docx]

**S1 File**

**Description of the EPIONCHO model, equations, calibration, modifications to incorporate vaccination and model vaccine efficacy, and results under varying assumptions of coverage and age groups targeted**

**Text A. Model Description**

EPIONCHO is a population-based, deterministic model that comprises a number of partial differential equations describing the population dynamics of *Onchocerca volvulus* with host age and time and the impact on these dynamics of control interventions. The system of partial differential equations is based on a host age- and sex-structured onchocerciasis dynamics model [[1-3](#_ENREF_1)]. These equations describe, respectively, the rate of change with respect to time and host age of the numbers of non-fertile, *N*, and fertile, *F*, adult female worms per host; of microfilariae per milligram of skin, *M*, and of infective (L3) larvae, *L*, per blackfly vector. The model has been previously modified to incorporate the effects of ivermectin treatment and treatment compliance [[2](#_ENREF_2), [3](#_ENREF_3)]. Here, we modify the framework further to incorporate vaccination. Although in this paper we do not explore the effects of both ivermectin treatment and vaccination, we present the general framework which can be adapted to reflect the intervention(s) under scrutiny.

The host population (and subsequently the parasite population) is partitioned into different treatment groups according to how regularly they receive ivermectin treatment (a group who takes treatment every round; two groups who take treatment every other round alternately, and a fourth group of systematic non-compliers who never takes treatment) [[3](#_ENREF_3)]. These different compliance groups are denoted with subscript *d*, their proportion in the population by $\eta$, the host sex groups (men and women) with subscript *s*, their proportion in the population by *q*, the time since the last ivermectin treatment by *τ*, and host age by *a*. The model was further stratified into two *i* groups, those who receive the vaccine and those who do not receive it. Definitions and values of model parameters (for savanna *Onchocerca volvulus*–*Simulium damnosum s.s./S. sirbanum* in northern Cameroon) are given in Table S2. The equations (omitting time and age dependencies on the left terms for simplicity, and assuming a balanced worm sex ratio) are as follows,

**Text B. Model Equations**

$\begin{matrix} \frac{\partial N_{s,d,i}}{\partial t}+\frac{\partial N_{s,d,i}}{\partial a}=(\frac{1}{2}m\beta\Omega_{s}(a-p)\delta_{H}[L(t-p)]L(t-p)\exp(-\mu_{H}p))\left(V^{W}_{i}(t,a)\right) \\ +[\lambda_{0}+\lambda_{1}(\tau)]F_{s,d,i}(t,a)-(\varpi+\sigma_{W})N_{s,d,i}(t,a) \end{matrix}$ (S.1)

$\frac{\partial F_{s,d,i}}{\partial t}+\frac{\partial F_{s,d,i}}{\partial a}=\varpi N_{s,d,i}(t,a)-[\lambda_{0}+\lambda_{1}(\tau)+\sigma_{W}] F_{s,d,i}(t,a)$ (S.2)

(S.3)

$M_{s,d,i}(t,a)=\left(1-V^{M}_{i}(t,a)\right)M_{s,d,i}(t,a)$ (S.4)

$\frac{\partial L_{s,d,i}}{\partial t}+\frac{\partial L_{s,d,i}}{\partial a}=\beta\Omega_{s}(a) \delta_{V_{0}}M_{s,d,i}(t,a)-\sigma_{L}[M_{s,d,i}(t,a)] L_{s,d,i}(t,a)$ (S.5)

$L(t)=\sum_{s} \sum_{d} \sum_{i} q_{s}\eta_{d}\xi_{i}\int_{a} \rho(a) \Omega_{s}(a)L_{s,d,i}(t,a) da$ (S.6)

Further description of the model can be found in [[1-4](#_ENREF_1)].

The model can reflect pre-control infection levels in a range of hypo-, meso-, hyper- and highly hyperendemic (also known as holoendemic) onchocerciasis foci by varying the annual biting rate (ABR) of the simuliid vectors, i.e. the yearly number of blackfly bites received by a maximally exposed person in a community. In this report we explore scenarios in which the baseline microfilarial prevalence ranges from meso- to holoendemic (Table S1), and ivermectin is not distributed because of co-endemicity with *Loa loa* and risk of severe adverse events (see main text).

| **Table A. Summary of baseline (pre-control) modelled epidemiological scenarios** | | | | |
| --- | --- | --- | --- | --- |
| **Pre-control endemicity** | **Microfilarial prevalence in all ages** | **Annual biting rate (ABR)^§^** | **Annual transmission potential (ATP)**^¶^ | **Mean microfilarial intensity^*^ in all ages**  **(microfilariae /mg)** |
| Mesoendemic | 40% | 7,300 | 88 | 11.2 |
| Hyperendemic | 60% | 15,470 | 373 | 23.9 |
| Highly hyperendemic | 80% | 85,800 | 4,290 | 58.9 |
| ^§^Annual biting rate (ABR): the average number of *Simulium* bites to which a person is exposed during a whole year.^¶^ Annual transmission potential (ATP): the average number of infective larvae (L3) of *O. volvulus* potentially received during a whole year by a person exposed to the ABR; the model assumes perennial transmission. Both the ABR and ATP are for a proportion of vector blood meals of human origin equal to 0.3 as reported for Cameroon [[4](#_ENREF_4)]. ^*^Arithmetic mean number of microfilariae per mg of skin. | | | | |

**Text C. Modelling Vaccine Efficacy**

In the model the vaccine was assumed to have two effects:

- A percentage reduction in the number of incoming worms capable of developing into reproductively functional adults $V_{i}^{W}$(prophylactic).
- A percentage reduction in skin microfilarial load $V_{i}^{M}$(therapeutic).

We modelled a vaccination programme targeting one to five year olds in its first year, followed by continuous yearly vaccination of one year olds subsequently. Based on animal model data [[5-7](#_ENREF_5)], we assumed an initial prophylactic efficacy, , of 50%, and an initial therapeutic efficacy, , of 90% (although these values were varied in the sensitivity analysis). The initial prophylactic and therapeutic effects of the vaccine were assumed to wane at a set rate *D* (=1/mean duration of any protection),

(S.7)

, (S.8)

whereat yearly intervals (continuous yearly vaccination) for the vaccinated age groups and 0 otherwise.

| **Table B.** Definition and values of parameters and variables for the onchocerciasis population dynamics model EPIONCHO | | | |
| --- | --- | --- | --- |
| **Symbol** | **Definition of variables and parameters** | **Expression, average value and units** | **Ref.** |
| *Pertaining to human host* | | | |
|  | Mean number of non-fertile female adult worms per person at time (*t*) and age (*a*); subscripts *s*, *d* and *i* denote host sex, treatment compliance category and vaccine group | Equation (*S*.1) |  |
|  | Mean number of fertile female adult worms per person at time (*t*) and age (*a*); subscripts *s, d* and *i* as above | Equation (*S*.2) |  |
|  | Mean number of microfilariae per milligram of skin at time (*t*) and age (*a*); subscripts *s, d* and *i* as above | Equation (*S*.3) |  |
| $\delta_{H}[L(t)]$ | Proportion of L3 larvae developing to adult worms within the human host as a function of the number of infective larvae received per unit time | $\frac{\delta_{H_{0}}+\delta_{H\infty}c_{H}m\beta L(t)}{1+c_{H}m\beta L(t)}$ | [[1](#_ENREF_1)] |
| $\delta_{H_{0}}$ | Proportion of L3 larvae developing to adult worms within the human host when $m\beta L(t)\to0$ | 0.0854 | [[4](#_ENREF_4)] |
| $\delta_{H_{\infty}}$ | Proportion of L3 larvae developing to adult worms within the human host when $m\beta L(t)\to\infty$ | 0.00299 | [[8](#_ENREF_8)] |
| $c_{H}$ | Severity of transmission intensity-dependent parasite establishment within the human host | 5.86 x 10^–3^ yr per L3 larva | [[8](#_ENREF_8)] |
| $\mu_{H}$ | Per capita death rate of human hosts | 0.04 yr^–1^ | [[1](#_ENREF_1)] |
| $\sigma_{W}$ | Per capita death rate of adult worms | 0.1 yr^–1^ | [[4](#_ENREF_4)] |
| $\sigma_{M_{0}}$ | Per capita death rate of microfilariae in the absence of ivermectin treatment | 0.8 yr^–1^ | [[4](#_ENREF_4)] |
| $\varpi$ | Per capita rate at which untreated, non-reproducing female worms become fertile | 0.59 yr^–1^ | [[9](#_ENREF_9)] |
| $\lambda_{0}$ | Per capita rate at which untreated fertile female worms become non-fertile in the absence of ivermectin treatment | 0.33 yr^–1^ | [[9](#_ENREF_9)] |
| $\varepsilon_{d}$ | Rate of production of microfilariae per fertile female worm scaled by the total weight (in milligrams) of microfilariae-bearing skin | 1.1538 yr^–1^ | [[4](#_ENREF_4), [10](#_ENREF_10)] |
| $\psi_{d}(t)$ | The relative (proportional) change in female worm fertility resulting from repeated exposures to ivermectin in compliance group *d* (set to 1 in this paper for no change) |  | [[3](#_ENREF_3)] |

| **Table B. Continued** | | | | | | |
| --- | --- | --- | --- | --- | --- | --- |
| **Symbol** | **Definition of variables and parameters** | | **Expression, average value and units** | | | **Ref.** |
| *Pertaining to human host (continued)* | | | | | | |
| $a_{m}$ | Maximum recorded human age in the reference population of northern Cameroon | | | 80 yr | [[1](#_ENREF_1)] | |
| *p* | Pre-patent period (from infection with L3 larvae to presence of detectable microfilariae in the skin) | | | 2 yr | [[4](#_ENREF_4)] | |
|  | Probability density function of host age *a* (using a truncated exponential distribution of survival times) | | | $\frac{\mu_{H}\exp(-\mu_{H} a )}{1-\exp(-\mu_{H}a_{m})}$ | [[1](#_ENREF_1)] | |
| $\eta_{d}$ | Proportion of the host population in ivermectin compliance group *d* (not applicable in this paper) | | | – | [[3](#_ENREF_3)] | |
| $q_{s}$ | Proportion of the host population in sex group (females/males) *s* | | | 0.45/0.55 | [[1](#_ENREF_1)] | |
| $\lambda_{1}(\tau)$ | Excess per capita rate at which fertile females become non-fertile following ivermectin treatment (embryostatic effect), set to 0 in this paper | | | $32.4 \exp(-19.6\tau)$yr^–1^ | [[9](#_ENREF_9)] | |
| $\sigma_{M_{1}}(\tau)$ | Excess per capita death rate of microfilariae following ivermectin treatment (microfilaricidal effect), set to 0 | | | $\left( \tau+9.6\times10^{-3}\right)^{-1.25}$yr^–1^ | [[9](#_ENREF_9)] | |
|  | Initial prophylactic efficacy of the vaccine against incoming worms (at the time of completion of the whole vaccination course) | | | 50% (or 70%) | [[5-7](#_ENREF_5)] | |
| $V_{i}^{W}(t,a)$ | Vaccine efficacy against incoming worms at time (*t*), age (*a*) and vaccine group *i* | | | Equation (*S*.7) | This work | |
|  | Initial therapeutic efficacy of the vaccine against microfilarial load (at the time of completion of the whole vaccination course) | | | 90% (or 95%) | [[5-7](#_ENREF_5)] | |
| $V_{i}^{M}(t,a)$ | Vaccine efficacy against microfilarial load at time (*t*), age (*a*) and vaccine group *i* | | | Equation (*S*.8) | This work | |
| *D* | Rate of decay of the protective effect of the vaccine (varied in sensitivity analysis) | | | 1/assumed mean duration of any protective effect of the vaccine (i.e. 1/20 yr = 0.05 yr^-1^) | [[11](#_ENREF_11)] | |
| $\xi_{i}$ | Coverage of vaccination | | | 80% | [[11](#_ENREF_11), [12](#_ENREF_12)] | |
| **Table B. Continued** | | | |  |  | |
| **Symbol** | | **Definition of variables and parameters** | | **Expression, average value and units** | **Ref.** | |
| *Pertaining to simuliid vector* | | | | | | |
|  | | Mean number of infective larvae per fly at time (*t*) | | Equation (*S*.6) |  | |
| $m$ | | Vector to host ratio | | ABR/$\beta$ |  | |
| $\beta$ | | Biting rate per fly on humans assuming a human blood index = 0.3 (for Cameroon) | | 31.2 yr^–1^ | [[1](#_ENREF_1), [8](#_ENREF_8)] | |
| $\delta_{V_{0}}$ | | Proportion of ingested microfilariae developing to the infective stage within the vector, per bite | | 0.005 | [[1](#_ENREF_1)] | |
| $\sigma_{L}[M_{s,d,i}(t,a)]$ | | Per capita net rate of loss of L3 larvae from vectors | | $(a_{H}/g)+\sigma_{L_{0}}+\mu_{V}+\alpha_{V}M_{s,d,i}(t,a)$ | [[4](#_ENREF_4)] | |
| $a_{H}$ | | Proportion of infective, L3 larvae shed per bite | | 0.5 | [[4](#_ENREF_4)] | |
| $g$ | | Average duration between consecutive blood-meals | | 0.0096 yr | [[4](#_ENREF_4)] | |
| $\sigma_{L_{0}}$ | | Per capita death rate of L3 larvae within the vector | | 104 yr^–1^ | [[4](#_ENREF_4), [8](#_ENREF_8)] | |
| $\mu_{V}$ | | Per capita death rate of uninfected blackflies | | 52 yr^–1^ | [[4](#_ENREF_4), [8](#_ENREF_8)] | |
| $\alpha_{V}$ | | Parasite-induced death rate of infected blackflies | | 0.6 yr^–1^ per microfilaria | [[4](#_ENREF_4)] | |
|  | | Age- and sex-specific measure of exposure to vectors | | $\left\{\begin{matrix} E_{s}\gamma_{s}E_{0}, a<a' \\ E_{s}\gamma_{s}\exp[-\alpha_{s}(a-a')], a>a' \end{matrix}\right.$ | [[1](#_ENREF_1)] | |
| $E_{s}$ | | Sex-specific exposure to vector bites (females/males) | | 0.90/1.08 | [[1](#_ENREF_1)] | |
| $E_{0}$ | | Fraction of exposure at age 0 in relation to that at age *a*’ from which exposure is allowed to change with age | | 0.10 | [[1](#_ENREF_1)] | |
| $\gamma_{s}$ | | Normalisation factors to ensure that the distribution of bites among age groups sums to 1 (females/males) | | 0.548/1.154 | [[1](#_ENREF_1)] | |
| $\alpha_{s}$ | | Age-specific change in contact rate with vectors for human hosts of sex *s* (females/males) | | –0.023/0.007 | [[1](#_ENREF_1)] | |

| **Table C.** Definition and values of parameters for mating probability and microfilarial prevalence calculations | | | |
| --- | --- | --- | --- |
| **Symbol** | **Definition of variables and parameters** | **Expression, average value and units** | **Ref.** |
|  | Mating probability at time (*t*), age (*a*), sex *s*, treatment compliance group *d*, and vaccine group *i* | $1-\left[1+\frac{W_{s,d,i}(t,a)}{k_{W}}\right]^{ -(k_{W}+1)}$ | [[13](#_ENREF_13)] |
| $W_{s,d,i}(t,a)$ | Mean number of female adult worms per person at time (*t*) and age (*a*)*,* *s, d and i* as above |  |  |
| $k_{W}$ | Inverse measure of degree of overdispersion in the distribution of worms among hosts | 0.35 | [[14](#_ENREF_14)] |
| $\pi_{d}(t)$ | Microfilarial prevalence at time *t* in treatment compliance group *d* | $\pi_{d}(t)=1-\left\{1+\frac{M_{d}(t)}{k_{M}[M_{d}(t)]}\right\}^{-k{}_{M}[M_{d}(t)]}$ |  |
| $k_{M}[M_{d}(t)]$ | Inverse measure of the degree of overdispersion in the distribution of skin microfilariae among hosts of ivermectin compliance group *d*, as a function of the mean microfilarial load | $k_{M}\left[M_{d}(t)\right]=\frac{k_{0} M_{d}(t)}{1+k_{1} M_{d}(t)}$ | [[15](#_ENREF_15)] |
| $k_{0}$ | Parameters of the relationship between $k_{M}$and skin microfilarial load | 0.013 | [[15](#_ENREF_15)] |
| $k_{1}$ |  | 0.025 | [[15](#_ENREF_15)] |

| **Table D. Long-term impact of vaccination on onchocerciasis annual transmission potential and microfilarial load in the absence of ivermectin treatment under different assumptions of the vaccine coverage** | | | | | |
| --- | --- | --- | --- | --- | --- |
|  | Years after vaccination | | | | |
|  | Pre-Control | 5 years | 10 years | 15 years | 30 years |
| Annual transmission potential (ATP) and percent reduction from baseline (%) | | | | | |
| Mesoendemic | 88 | 79  (10%) | 75  (14%) | 72  (18%) | 64  (28%) |
| Hyperendemic | 373 | 336  (10%) | 323  (13%) | 313  (16%) | 294  (21%) |
| Highly hyperendemic | 4,365 | 3,923  (10%) | 3,766  (14%) | 3,645  (16%) | 3,426  (22%) |
| Mean microfilarial load (arithmetic mean no. microfilariae/mg, all ages) and percent reduction (%) | | | | | |
| Mesoendemic | 11.2 | 9.6  (14%) | 9.0  (20%) | 8.5  (24%) | 7.4  (34%) |
| Hyperendemic | 24.0 | 20.6  (14%) | 19.5  (19%) | 18.6  (22%) | 17.3  (28%) |
| Highly hyperendemic | 59.2 | 50.8  (14%) | 47.9  (19%) | 45.6  (23%) | 41.9  (29%) |
| *Model assumes an initial vaccine efficacy against the development of incoming worms of 50% and against skin microfilarial load of 90%. Results assume mean duration of protective and therapeutic effects of 20 years (rate of decay = 0.05 per year),* *vaccination programme targeting initially one-five year olds with continuous vaccination of one year olds after the first year of the programme and an 60% coverage of vaccinatio*n*. Annual transmission potential (ATP): the average number of L3 larvae potentially received per person per year.* | | | | | |

| **Table E. Long-term impact of vaccination on onchocerciasis annual transmission potential and microfilarial load in the absence of ivermectin treatment under different assumptions of the targeted age group** | | | | | |
| --- | --- | --- | --- | --- | --- |
|  | Years after vaccination | | | | |
|  | Pre-Control | 5 years | 10 years | 15 years | 30 years |
| Annual transmission potential (ATP) and percent reduction from baseline (%) | | | | |  |
| Mesoendemic | 88 | 78  (11%) | 74  (16%) | 71  (20%) | 63  (29%) |
| Hyperendemic | 373 | 332  (11%) | 320  (14%) | 310  (17%) | 292  (22%) |
| Highly hyperendemic | 4,365 | 3,879  (11%) | 3,731  (15%) | 3,618  (17%) | 3,410  (22%) |
| Mean microfilarial load (arithmetic mean no. microfilariae/mg, all ages) and percent reduction (%) | | | | | |
| Mesoendemic | 11.2 | 9.4  (16%) | 8.8  (22%) | 8.3  (26%) | 7.2  (35%) |
| Hyperendemic | 24.0 | 20.2  (16%) | 19.1  (20%) | 18.3  (24%) | 17.0  (29%) |
| Highly hyperendemic | 59.2 | 49.8  (16%) | 47.0  (21%) | 44.9  (24%) | 41.3  (30%) |
| *A: Model assumes an initial vaccine efficacy against the development of incoming worms of 50% and against skin microfilarial load of 90%. Results assume mean duration of protective and therapeutic effects of 20 years (rate of decay = 0.05 per year),* *continuous vaccination of five year olds and an 80% coverage of vaccinatio*n*. Annual transmission potential (ATP): the average number of L3 larvae potentially received per person per year.* | | | | | |

**References**

1. Filipe JAN, Boussinesq M, Renz A, Collins RC, Vivas-Martinez S, Grillet ME, Little MP, Basáñez MG. Human infection patterns and heterogeneous exposure in river blindness. *Proc Natl Acad Sci U S A.* 2005;102(42): 15265-15270.

2. Churcher TS, Basáñez MG. Density dependence and the spread of anthelmintic resistance. *Evolution.* 2008;62(3): 528-537.

3. Turner HC, Churcher TS, Walker M, Osei-Atweneboana MY, Prichard RK, Basáñez MG. Uncertainty surrounding projections of the long-term impact of ivermectin treatment for human onchocerciasis. *PLoS Negl Trop Dis.* 2013;7(4): e2169.

4. Basáñez MG, Boussinesq M. Population biology of human onchocerciasis. *Philos Trans R Soc Lond B Biol Sci.* 1999;354(1384): 809-826.

5. Babayan SA, Allen JE, Taylor DW. Future prospects and challenges of vaccines against filariasis. *Parasite Immunol.* 2012;34(5): 243-253.

6. Hess JA, Zhan B, Bonne-Année S, Deckman JM, Bottazzi ME, Hotez PJ, Klei TR, Lustigman S, Abraham D. Vaccines to combat river blindness: expression, selection and formulation of vaccines against infection with *Onchocerca volvulus* in a mouse model. *Int J Parasitol.* 2014; 44(9): 637-646.

7. Arumugam S, Wei J, Ward D, Abraham D, Lustigman S, Zhan B, Klei TR. Vaccination with a genetically modified *Brugia malayi* cysteine protease inhibitor-2 reduces adult parasite numbers and affects the fertility of female worms following a subcutaneous challenge of Mongolian gerbils (*Meriones unguiculatus*) with *B. malayi* infective larvae. *Int J Parasitol.* 2014;44(10): 675-679.

8. Basáñez MG, Collins RC, Porter CH, Little MP, Brandling-Bennett D. Transmission intensity and the patterns of *Onchocerca volvulus* infection in human communities. *Am J Trop Med Hyg.* 2002; 67(6): 669-679.

9. Basáñez MG, Pion SDS, Boakes E, Filipe JAN, Churcher TS, Boussinesq M. Effect of single-dose ivermectin on *Onchocerca volvulus*: a systematic review and meta-analysis. *Lancet Infect Dis.* 2008; 8(5): 310-322.

10. Duke BOL. The population dynamics of *Onchocerca volvulus* in the human host. *Trop Med Parasitol.* 1993;44(2): 61-68.

11. Chan MS, Woolhouse ME, Bundy DAP. Human schistosomiasis: potential long-term consequences of vaccination programmes. *Vaccine.* 1997;15(14): 1545-1550.

12. Immunization, measles (% of children aged 12-23 months). 2015. Available: [<http://data.worldbank.org/indicator/SH.IMM.MEAS?order=wbapi_data_value_2011+wbapi_data_value+w>]. Accessed 23^rd^ June 2015.

13. May RM. Togetherness among schistosomes: its effects on the dynamics of the infection. *Math Biosci.* 1977;35(3–4): 301-343.

14. Bottomley C, Isham V, Collins RC, Basáñez, MG. Rates of microfilarial production by *Onchocerca volvulus* are not cumulatively reduced by multiple ivermectin treatments. *Parasitology.* 2008;135(13): 1571-1581.

15. Turner HC, Walker M, Churcher TS, Basáñez MG. Modelling the impact of ivermectin on River Blindness and its burden of morbidity and mortality in African savannah: EpiOncho projections. *Parasit Vectors.* 2014;7: 241.
